# Engineered Nanoparticles, Natural Nanoclay and Biochar, as Carriers of Plant-Growth Promoting Bacteria

**DOI:** 10.3390/nano12244474

**Published:** 2022-12-17

**Authors:** Milica Pavlicevic, Wael Abdelraheem, Nubia Zuverza-Mena, Tana O’Keefe, Salma Mukhtar, Gale Ridge, John Ranciato, Christy Haynes, Wade Elmer, Joseph Pignatello, Luca Pagano, Marina Caldara, Marta Marmiroli, Elena Maestri, Nelson Marmiroli, Jason C. White

**Affiliations:** 1 Department of Chemistry, Life Sciences and Environmental Sustainability, University of Parma, 43124 Parma, Italy; 2 Connecticut Agricultural Experimental Station, New Haven, CT 06511, USA; 3 Chemistry Department, Faculty of Science, Sohag University, Sohag 82524, Egypt; 4 Department of Chemistry, University of Minnesota, Minneapolis, MN 55455, USA; 5 Interdepartmental Center SITEIA.PARMA, University of Parma, 43124 Parma, Italy; 6 National Interuniversity Consortium for Environmental Sciences (CINSA), 43124 Parma, Italy

**Keywords:** nanoclay, mesoporous silica, biochar, PGPR, nitrogen, phosphorus

## Abstract

The potential of biochar and nanoparticles to serve as effective delivery agents for beneficial bacteria to crops was investigated. Application of nanoparticles and biochar as carriers for beneficial bacteria improved not only the amount of nitrogen-fixing and phosphorus-solubilizing bacteria in soil, but also improved chlorophyll content (1.2–1.3 times), cell viability (1.1–1.5 times), and antioxidative properties (1.1–1.4 times) compared to control plants. Treatments also improved content of phosphorus (P) (1.1–1.6 times) and nitrogen (N) (1.1–1.4 times higher) in both tomato and watermelon plants. However, the effect of biochars and nanoparticles were species-specific. For example, chitosan-coated mesoporous silica nanoparticles with adsorbed bacteria increased the phosphorus content in tomato by 1.2 times compared to a 1.1-fold increase when nanoclay with adsorbed bacteria was applied. In watermelon, the situation was reversed: 1.1-fold increase in the case of chitosan-coated mesoporous silica nanoparticles and 1.2 times in case of nanoclay with adsorbed bacteria. Our findings demonstrate that use of nanoparticles and biochar as carriers for beneficial bacteria significantly improved plant growth and health. These findings are useful for design and synthesis of novel and sustainable biofertilizer formulations.

## 1. Introduction

Given the relatively low efficiency and high environmental impact of conventional agrochemicals [1,2,3,4], novel strategies are needed to provide plants with sufficient nutrients and protection from pests and pathogens while simultaneously reducing negative environmental impacts. Commonly used fertilizers have relatively low efficiency due to nutrient leaching, volatilization or precipitation [3,5]. Furthermore, their environmental impact is detrimental, with application leading to eutrophication, increased soil salinity, and air pollution as a result of nitrate accumulation and changes in the rhizosphere that can directly impact crop quality [4,6]. Given these negative consequences of “conventional” agriculture, the United Nations (UN) actively supports sustainable agriculture in order to achieve their sustainable development goals of “zero hunger”, lower food waste, and increased food quality and security [7].

Biochar is a product of thermochemical transformation of plant (or animal) biomass, and its addition to soil can increase the content of carbon, nitrogen and phosphorus in soil, minimize the phytotoxic effects of contaminated soil, stimulate the soil microbial community, and improve growth and yield [8,9,10,11]. On a molecular level, biochar can be viewed as an engineered nanocomposite [12,13,14], which allows for direct comparison of effect of nanoparticles and biochar. As noted above, biochar can be important agricultural amendment. Additionally, unlike “conventional” fertilizers, the addition of biochar to soil has minimal negative impact on plants and the environment and can also enable plant growth in soils that are considered marginal or not suitable for agricultural use (e.g., sandy, loamy, acidic, or contaminated soils) [15,16,17,18]. Given its porosity, adsorbent ability and relatively high nutrient content, biochar inoculated with microorganisms has been found to improve plant growth, yield, stress tolerance, and the adsorption of macro- and micronutrients [15,16,17,18,19]. However, biochars often differ significantly in their properties and performance, with their functionality also being impacted by soil characteristics such as pore size, pH, and moisture content. In addition, the selection of type(s) of microorganism(s) as the inoculant is dictated by specific needs of the plant (e.g., phosphorus “capture”, nitrogen adsorption, protection against pathogens, etc.) [16,19,20,21]. For example, Husna et al. [16] found that coconut shell biochar with moisture content of 26.86%, pH 7.74 and average pore size 6.59 μM increased the survivability of inoculated phosphate solubilizing microorganism up to 6 months. Egamberdieva et al. [20] found that biochar produced by hydrothermal carbonization at 210 °C and inoculated with Pseudomonas putida or Stenotrophomonas pavanii reduced root rot in narrow-leafed lupin (Lupinus angustifolius L.). Species from the Rhizobium genus inoculated on hydrochar and biochar obtained from pinewood at 600 °C showed potential to increase nitrogen absorption and plant growth in a sandy soil [21,22]. In addition, Hansen et al. [23] found that addition of gasification biochar had a positive effect on the population of microorganisms in the soil, while at the same time improving soil quality, increasing potassium (K) bioavailability and modulating soil pH. However, Yang et al. [24] reported that effect of gasification biochar on soil microbial community was strongly dependent on starting material from which biochar was derived. In fact, Gram (+) and Gram (−) bacteria and fungi were all affected by biochar amendment to different extents. As a delivery system, gasification biochar has been less examined than hydrochars or biochars produced by pyrolysis. However, Sun et al. [25] found that biochar produced by gasification and inoculated with rhizobia had more pronounced positive effects on nitrogen content and growth of black locust (*Robinia pseudoacacia*) seedlings than did biochar produced by pyrolysis and inoculated with the same concentration of rhizobia. In the same way, Graziano et al. [26] found positive effects on soil and plants when working with wheat and maize.

Nanoparticles, defined as particles within the size range of 1–100 nm, when used appropriately can offer protective effects for plants (acting as a pesticides and nutrients), promote plant growth, aid in nutrient absorption, and in the form of hydrogels can increase the efficiency of water management [27,28,29,30]. Beside these advantages of nanoparticles, they can also be added in smaller quantities than conventional agrochemicals and will enable slow and controlled release of nutrients [31,32]; thus, potential negative impacts on the environment can be significantly reduced. Importantly, there are still many unanswered questions with regard to safety and sustainability of nanoparticles [30,31,32,33]. Although nanoparticles have been utilized in medicine as drug delivery systems for some time, their use as delivery systems in agriculture is still developing [34,35]. For example, Buchman et al. [36] used chitosan-coated mesoporous silica to modulate the expression of stress-related genes in watermelon and minimize the impacts of fungal infection. Additionally, mesoporous silica nanoparticles, due to their porous structure and high loading capacity, have been successfully used for gene, drug, and pesticide delivery [37,38,39]. Nanoclays also have a high loading potential due their large surface area and cation exchange capacity [40,41]. An additional advantage of using both mesoporous silica and nanoclays as carriers is that they both contain silicon, and as noted above, this element has been linked with increased resistance to pathogens due to its capacity to activate antioxidant defenses [42,43].

Plant-growth promoting rhizobacteria (PGPR) are soil microbes that provide a series of benefits for the host plant, largely due to a wide range of signaling molecules “traveling” to and from a plant’s root system [44]. Plant-growth promoting rhizobacteria stimulate plant growth through enhanced acquisition of nutrients, increase plant immunity in response to root exudates, and provide enhanced protection through interference with pathogen toxin production [44,45]. *Azetobacter vinaldii* is a rod-shaped, obligate aerobic bacterium, originally first isolated from soil in Vineland, New Jersey, United States [46,47]. This bacterium possesses 3 versions of the enzyme nitrogenase that allows robust participation in the process of “nitrogen fixation”, e.g., conversion of nitrogen (N2) from the air into ammonia (NH_3_) [46,47]. *Bacillus megaterium* was isolated from different types of habitats and is primarily considered to be a soil bacterium [48,49]. In addition to its industrial use for production of different types of exoenzymes [48], *Bacillus mageterium* has well-documented phosphorus-solubilizing ability, e.g., capability to convert insoluble forms of phosphorus into phosphorus-containing compounds that could be used by plants (by secreting enzymes phosphatases and phytases) [49,50].

The aim of this work was to investigate the effects of biochars produced by different methodologies (gasification, pyrolysis and pyrogasification) and the effects of silica-containing nanoparticles (mesoporous silica and nanoclays) as carriers for PGPR in two plant species: tomato and watermelon. We were interested in assessing not only the impact of these two types of materials on nutrient content, viability, and antioxidant properties of plants, but also their individual potential to act as delivery systems for beneficial bacteria. Importantly, although nanoparticles and biochar represent two separate but equally complex material types, both were found to serve as excellent niches for protection and growth of PGPR.

The results of this study contribute to our understanding of potential of biochar, nanoparticles and PGPR as fertilizers and plants’ immune enhancers to increase agricultural productivity.

## 2. Materials and Methods

### 2.1. Chemicals

2,2-diphenyl-1-picrylhydrazyl (DPPH), 2,2’-azino-bis(3-ethylbenzothiazoline-6-sulfonic) acid (ABTS), nanoclay (hydrophilic bentonite), 6-hydroxy-2,5,7,8-tetramethylchroman-2-carboxylic acid (Trolox), L-ascorbic acid, tetraethylorthosilicate (TEOS), cetyltrimethylammonium bromide (CTAB), and chlorotrimethylsilane were purchased from Sigma-Aldrich (St. Louis, MO, USA). 3-(4,5-dimethylthiazol-2-yl)-2,5-diphenyltetrazolium bromide (MTT) and Dulbecco’s Modified Eagle’s Limiting Medium were purchased from Fisher Scientific (Waltham, MA, USA). 2-[methoxy-(polyethyleneoxy) 9−12 propyl]-trimethoxysilane was purchased from Gelest (Morrisville, PA, USA). Chitosan was purchased from Spectrum (New Brunswick, NJ, USA). Pikovskaya medium was purchased from HiMedia Laboratories (West Chester, PA, USA), while nutrient agar was purchased from Fisher Scientific (Waltham, MA, USA). *Azotobacter vinelandii* (strain designation: DSM 2289; ATCC 478; VKM B-1617) and *Bacillus megaterium* (strain designation: DSM 32; VKM B-512) were purchased from ATCC (Manassas, VA, USA) and kept at −80 °C prior to analysis. These strains of bacteria were selected due to their confirmed efficiency in nitrogen-fixation and phosphorus solubilization, respectively. Naked biochar was purchased from American Biochar Company (Niles, MI, USA). Aries Green biochar was purchased from Aries Clean Technologies LLC (Franklin, TN, USA). The remaining chemicals were purchased from Merck Milipore (Burlington, MA, USA).

### 2.2. Synthesis of Chitosan-Coated Mesoporous Silica Nanoparticles (MSN) and Characterization of Nanoclay (NC) and MSN

Synthesis of chitosan-coated mesoporous silica was performed according to Buchman et al. [36], except that the ultracentrifugation speed was reduced to 21,500 g (from 61,579 g). Additionally, the elemental analysis of nanoclay was determined by inductively coupled plasma optical emission spectrophotometry (ICP-OES) (iCAP 6000 series, Thermo Fisher Scientific, Waltham, MA, USA). Prior to ICP-OES analysis, 0.1 g of nanoclay was weighed and digested with 3 mL of 68% nitric acid for 45 min at 115 °C (DigiPrep MS, SCP Science, Cham-plain, NY, USA). The sample was then diluted to 15 mL with distilled water and was allowed to “settle” overnight. The size of nanoparticles was determined by Transmission Electron Microscopy (TEM) (HT7800 TEM, Hitachi, Japan). The surface area and pore volume of NC and MSN were determined by nitrogen physisorption (Autosorb IQ, Quantachrome Instruments, Boynton Beach, FL, USA). The hydrodynamic diameter and ζ potential of the nanoparticles were determined by a zeta sizer (Nano-ZS90, Malvern Pananalytical, Malvern, UK). Prior to measurements, samples were sonicated 30 min at ambient temperature. The concentration of both nanoparticles for analyses was 0.5 mg/mL. Additionally, to exclude the presence of impurities in nanoclay, elemental analysis using energy dispersive X-ray spectroscopy (EDX) was done. Elemental analysis by EDX was performed by dispersing nanoclay particles in 95% ethanol and mounting them in a carbon-coated Ni grid. The analysis was performed with an EDX detector (80T, Oxford Instruments) attached to a Hitachi 7800 transmission electron microscope (TEM).

### 2.3. Characterization of Biochars

Aries green biochar (AB) was derived from wood waste via downdraft gasification and the naked biochar (NB) was derived from recycled wood through complete pyrolysis. A third biochar sample (in this paper “Italian biochar”—IB) was produced from wood pellets in a prototype pyrogasification system <50 kW (this is the same biochar named A4 in Marmiroli et al. [51]).

Measurement of pH and electrical conductivity (EC) of biochars were conducted according to Dume [52]. Briefly, biochars’ pH and electrical conductivity were measured in distilled water at 1:10 biochar to water ratio (*m*/*v*) after shaking for 30 min. Samples were left to “settle” 10 min prior to measurements.

The cation exchange capacity (CEC) was determined as described by Batista et al. [53]. Briefly, 2 g of sample was mixed with 100 mL of 0.5 mol/L HCl. The flask was then closed and shaken at 150 rpm for 30 min at ambient temperature. Excess acidic aqueous solution was then removed by vacuum and the material was washed twice with 50 mL portions of deionized water containing a few drops of 1% (*m*/*v*) AgNO3. The sample was then transferred to a new Erlenmeyer flask, and 100 mL of 0.35 mol/L (CH3COO)_2_Ba was added and stirred on a magnetic stirrer for 15 min (ambient temperature). The material was then filtered and washed 3 times with 100 mL portions of water. The solid was discarded and the filtrate was titrated against 0.1 mol/L NaOH solution, using 5 drops of phenolphthalein as indicator. The CEC was calculated using following equation:

$$\mathrm{CEC}=\frac{V\left(\mathrm{mL}\right)\times 0.1\frac{\mathrm{mol}}{L}\left(\mathrm{NaOH}\right)\times 100}{2g}$$

The surface area and pore volume of biochars were determined via nitroge3n physisorption (Autosorb IQ, Quantachrome Instruments, Boynton Beach, FL, USA). Hydrodynamic diameter and ζ potential of biochars were determined by zeta sizer (Nano-ZS90, Malvern Pananalytical, Malvern, UK). Prior to measurements samples were sonicated 30 min at room temperature. Concentration of samples was the same as for nanoparticles (0.5 mg/mL).

Elemental analysis of biochars was determined by inductively coupled plasma optical emission spectrophotometry (iCAP 6000 series, Thermo Fisher Scientific, Waltham, MA, USA). Prior to ICP-OES analysis, 0.1 g of homogenized sample was digested with 3 mL of 68% nitric acid for 45 min at 115 °C (DigiPrep MS, SCP Science, Champlain, NY, USA). The sample was then diluted to 15 mL with distilled water and was allowed to “settle” overnight.

The content of nitrogen (N) was determined on a nitrogen analyzer (FP628, LECO, St. Joseph, MI, USA). Briefly, 0.1 g of sample was measured and closed in aluminum foil (provided with the instrument). The analytical program settings included cellulose standard, EDTA standard, and an Association of American Feed Control Officials (AAFCO) standard (also provided with the instrument) that were used as negative and positive controls, respectively.

### 2.4. Characterization of Soil Substrate and Fertilizer

Promix BX (Premier Hort Tech, Quakertown, PA, USA) was used as the soil substrate. The pH was determined according to Environmental Protection Agency (EPA) method 9045D. Briefly, a 1:1 (*m*/*v*) of substrate: distilled water was mixed for 5 min on magnetic stirrer and left to “settle” for 1 h. The content of phosphorus (P) in the substrate was determined by ICP-OES (iCAP 6000 series, Thermo Fisher Scientific, Waltham, MA, USA). Prior to ICP-OES analysis, 0.1 g of homogenized sample was digested with 3 mL of 68% nitric acid for 45 min at 115 °C (DigiPrep MS, SCP Science, Champlain, NY, USA). The sample was then diluted to 15 mL with distilled water and left to “settle” overnight. The content of nitrogen (N) was determined by a nitrogen analyzer (FP628, LECO, St. Joseph, MI, USA). Miracle-Gro all-purpose fertilizer (Marysville, OH, USA) was used in the experiment. The content of phosphorus and nitrogen in the substrate was determined as described above.

### 2.5. Adsorption of Bacteria on Nanoparticles and Biochars, Determination of Loading Efficiency and Sample Characterization

The adsorption of bacteria to the nanoparticles was done via a modified procedure described by Deng et al. [54]. In short, in flasks were prepared containing 50 mL of autoclaved distilled water, 0.1 g nanoparticles (chitosan-coated mesoporous silica or nanoclay), and 2 mL of mixture containing 1 mL of 2 × 108 CFU/mL of *A. vinelandii* and 1 mL 2 × 108 CFU/mL *B. megaterium* was added. Bacteria were grown on nutrient agar (Fisher Scientific, Waltham, MA, USA) at 28 °C for 48 h. The mixture was then shaken for 6 h at 6000 rpm at ambient temperature, centrifuged at 10,000 rpm for 10 min and the recovered pellet was air-dried under a hood. The number of loaded bacteria was determined by a modified procedure described by Deng et al. [54]. One ml of supernatant from the previous step was grown on nutrient agar (28 °C, 48 h) and the CFU/mL is determined. The number of loaded bacteria was calculated by difference between initial CFU and CFU after adsorption. The loading of bacteria was verified using a Scanning Electron Microscopy (SEM) (TM3030 Plus, Hitachi High-Tech Group, Japan). Sample preparation for SEM analysis included: the sample holder was cleaned with alcohol, dried and carbon tape was placed in the middle of the holder. A small amount of powdered, homogenized sample was removed with sterilized spatula, placed on carbon tape, pressed lightly with sterilized tweezers (blunt end), after which excess was taped off on filter paper. SEM images were taken at D6.1 x 180 in back scattered electron (BSE) mode and under energy dispersive x-ray spectroscopy (EDX) observational conditions. Additionally, TEM images of nanoparticles with adsorbed bacteria were taken (HT7800 TEM, Hitachi, Japan) using 2.5% glutaraldehyde in phosphate-buffered saline for fixation of bacteria. The samples were left to dry at room temperature overnight prior to imaging.

For adsorption on biochars, the preparation of biochar was according to Husna et al. [16]. In short, 30 g of biochar was ground and sieved to 1 mm and then autoclaved for 1 h at 121 °C. The loading of bacteria was verified using scanning electron microscopy (SEM) (TM3030 Plus, Hitachi High-Tech Group, Japan). Inoculation of bacteria on biochar was also done as described by Husna et al. [16]. Two ml of consortium (1 mL 2 × 108 CFU/mL of *A. vinelandii* and 1 mL 2 × 108 CFU/mL of *B. megaterium*) and 11 mL of distilled/autoclaved water were mixed and applied onto the biochar using a sterile syringe. The biochar was then sealed in a sterile bag and left at ambient temperature for 24 h. Sample preparation and working parameters were the same as for the nanoparticles.

### 2.6. Plant Growth Experimental Design

Tomato (*Solanum lycopersicum*, cultivar Bonny Best; Totally Tomato, Randolph, WI, USA) and watermelon (*Citrullus lanatus*, cultivar Sweet Baby; Harris Seed Co., Rochester, NY, USA) were grown in a greenhouse in pots filled with 250 mL of soil. The concentration of nanoparticles in the soil was 250 mg/L, while the concentration of biochars in the soil was 100 mg/L. Mixing of either nanoparticles or biochars with soil was done in 2 L sterile bags. For samples marked as BAC, 2 mL of consortium (1 mL 2 × 108 CFU/mL of *A. vinelandii* and 1 mL 2 × 108 CFU/mL of *B. megaterium*) was added directly into the soil (in the same sterile bag) and mixed thoroughly by shaking. In total there were 12 treatments as described in Table 1. The arrangement of plants was randomized and each treatment had 8 replicates.

This study consisted of three experiments. Tomato was used in experiments 1 and 2. Watermelon was grown in experiment 3. Half the recommended dose of Miracle-Gro (1.88 g/kg) was applied in experiments 2 and 3 once per week (10 mL per plant). In the 1st experiment no fertilizer was added. All plants were grown for three weeks before being transplanted into pots with 250 mL of soil substrate with the various amendments. Measurements of P and N content, soil pH, and physiological parameters, as well as microbial analyses, were performed at 0, 7, 14, and 28 days after transplanting (DAT) in tomato, and 0, 10, 20, and 30 DAT in watermelon.

### 2.7. Determination of Soil pH after Treatment

The soil pH after harvest was determined using the same procedure as for the soil substrate (Section 2.4.). Briefly, 1:1 (*m*/*v*) of substrate: distilled water was mixed for 5 min on a magnetic stirrer and left to “settle” for 1 h before measurement.

### 2.8. Extraction and Characterization of Bacteria Populations

The extraction of bacteria from the soil was performed as described by Fox et al. [55] with modification. Briefly, 3 g of soil was mixed with 20 mL of sterile NaCl [0.85% (*w*/*v*)] solution for 30 min at 75 rpm at 4 °C. The suspensions were then left to settle for 1 h. Three aliquots (0.5 mL each) were then taken: one was used for determination of nitrogen-fixing bacteria; the others for the determination of phosphorus-solubilizing bacteria. These aliquots were serially diluted (by 10 folds) in 0.85% saline and colony forming units (CFU)/mL were determined by a plate counting method. Bacteria were grown at 28 °C for 48 h. Total bacteria were grown on nutrient agar (Fisher Scientific, Waltham, MA, USA), nitrogen-fixing bacteria were grown on nitrogen-free media (NFM) (prepared according to Dobereiner [56], with 15 g of agar added per 1 L to obtain solid media), and phosphorus-solubilizing bacteria were grown on Pikovskaya agar (HiMedia Laboratories, West Chester, PA, USA).

### 2.9. Determination of P and N Content in Plant Leaves

Samples were dried at 105 °C overnight and then ground. For determination of N content, 0.1 g of dried and homogenized sample was analyzed by a nitrogen analyzer (FP628, LECO, St. Joseph, MI, USA). To determine P content, 0.1 g of homogenized sample was digested with 3 mL of 68% nitric acid for 45 min at 115 °C (DigiPrep MS, SCP Science, Champlain, NY, USA). The sample was then diluted to 15 mL with distilled water and left to “settle” overnight. P content was then determined by ICP-OES (iCAP 6000 series, Thermo Fisher Scientific, Waltham, MA, USA).

### 2.10. Physiological Endpoints

Measurement of chlorophyll content was done spectrophotometrically as described by Li et al. [57]. Briefly, 0.1 g of fresh, homogenized sample was extracted with 50 mL of 95% ethanol (120 rpm, 1 h, room temperature). The pellet was discarded and supernatant was analyzed at 649 and 665 nm. The content of chlorophylls was calculated according to following formulas:

$$Chl\;a\;\left(\frac{\mathrm{mg}}{\mathrm{mL}}\right)=\frac{\left(12.7\times A665\right)-\left(2.69\times A649\right)}{1000}$$

$$Chl\;b\;\left(\frac{\mathrm{mg}}{\mathrm{mL}}\right)=\frac{\left(22.9\times A649\right)-\left(2.69\times A665\right)}{1000}$$

(1) Chl total=Chl a+Chl b

where *Chl a* is chlorophyll a, *Chl b* is chlorophyll b, *A*649 and *A*665 are absorbances measured at 649 and 665 nm, respectively, and *Chl* total is total chlorophyll content.

The formation of radical oxygen species was monitored by 2,2-diphenyl-1-picrylhydrazyl (DPPH) and 2,2’-azino-bis(3-ethylbenzothiazoline-6-sulfonic acid) (ABTS) assays. The DPPH assay was performed according to Sancez-Moreno et al. [58], whereas the ABTS assay was done as described by Re at al. [59]. Briefly, the extraction procedure for both tests was the same: samples were air-dried for 48 h under the hood. Then 0.125 g of dried, homogenized sample was extracted with 6.86 mL of 70% EtOH (120 rpm, 2 h, room temperature). For DPPH, 0.1 mL of sample was mixed with 1.9 mL of fresh DPPH solution (0.025 g/L DPPH in methanol), incubated for 30 min in the dark, and then absorbance was measured at 515 nm. The ABTS reagent was prepared 14 h earlier by mixing 5 mL of 7 mM ABTS solution (in water) with 5 mL of 2.45 mM potassium persulfate (in water) and kept in the dark at room temperature before use. Prior to testing, the ABTS reagent was diluted (with water) until A at 734 nm was between 0.65 and 0.75 (approximately 100×). For ABTS tests, 0.2 mL of sample was mixed with 1.8 mL of fresh ABTS solution, incubated for 30 min in the dark and absorbance was measured at 734 nm. As a standard for both ABTS and DPPH tests, Trolox was used at 500 μmol/L, 200 μmol/L, 100 μmol/L, 50 μmol/L, 25 μmol/L and 10 μmol/L. Trolox equivalents (TE) were determined from standard curves.

Plant cell viability was assessed by MTT assay as described by Shoemaker et al. [60]. Briefly, samples were air-dried for 48 h under a hood, and 0.375 g of dry, homogenized sample was extracted with 6.25 mL of distilled water at 100 °C for 45 min. After the solution had cooled, 0.5 mL of sample was pipetted to a new vial. The extract was then diluted with distilled water in the ratio 1:20. The MTT assay was performed by adding 400 μL of sample extract, 400 μL of 1 mM ascorbic acid (in water), 400 μL of Dulbecco’s modified Eagle’s medium, and 120 μL MTT (3 mg/mL in phosphate buffered saline), followed by incubation for 60 min at 37 °C. Absorbance was measured at 595 nm.

At the end of each experiment, stem length, total fresh mass, and fresh root mass were measured.

### 2.11. Statistical Analysis

All analyses were done in triplicate. A one-way analysis of variance (ANOVA) with repetition was used to assess difference between samples at different time points. Differences between means were determined by the Tukey test. Testing was done in SPSS software version 24 (IBM, Armonk, NY, USA). To test possible interactions between factors, a two-way ANOVA with repetition was also done in XLSTAT 2016 software (Addinsoft, NY, USA).

## 3. Results and Discussion

### 3.1. Synthesis of Chitosan-Coated Mesoporous Silica Nanoparticles (MSN) and Characterization of Nanoclay (NC) and MSN

The TEM micrographs demonstrate that MSN had rounded shape with the average diameter of 39 ± 8 nm (Figure S1), which is in agreement with Buchman et al. [36]. A much larger average diameter of 91 ± 7 nm was observed for nanoclay which possessed an irregular configuration (Figure S2). Due to the presence of free amino groups in chitosan coating [61], the ζ potential of MSN was positive: + 27.33 ± 0.59 mV. Conversely, the ζ potential of NC was negative: −39.35 ± 0.55 mV. The hydrodynamic diameter of the sample (1923.4 ± 7.8 nm) was nearly two-fold larger than that of the NC sample (823.13 ± 28.2 nm). This suggests that MSNs may aggregate even after extensive sonication. The lower zeta potential of MSN particles compared to NC particles explains their tendency to aggregate, regardless of charge type [62]. The results of the elemental analysis for the nanoclay with ICP-OES and EDX are shown in the Tables S1 and S2, respectively. The EDX mapping and signals arising from individual elements are shown on Figure S3. As evident from the both Tables S1 and S2 and Figure S3, the main elements detected in the nanoclay were silicon (Si), oxygen (O), aluminum (Al), iron (Fe), calcium (Ca), sodium (Na), magnesium (Mg), and potassium (K). These results correspond to that reported by Nam et al. [63] for bentonite nanoclay. Signals for nickel (Ni), carbon (C), copper (Cu), gold (Au), and cobalt (Co) were most likely “background signals” attributed to the grid and internal equipment components. The surface area and pore volume of MSN and NC are discussed below.

### 3.2. Characterization of Biochars

Table S3 shows the pH, conductivity and cation exchange capacity of the different biochars.

The “Italian” biochar (IB) had higher pH and electric conductivity (EC) compared to Aries Green biochar (AB) and Naked biochar (NB), whereas the cation exchange capacity (CEC) of IB was similar to that of AB. Singh et al. [64] reported that the pH of the feedstock is correlated to the EC, and both are influenced by the temperature of the biochar production process. Given that all three biochars were produced from wood residue, differences in pH and EC were more likely a reflection of different production methods. To further investigate the pH differences, elemental content of biochar was analyzed. All biochars had overall the same elemental composition, except on the content of main cations in biochar (calcium (Ca), sodium (Na), and potassium (K)) (Figure S4).

As shown in Figure S4., IB had significantly higher concentration of Ca and Na than AB and NB, while differences in K concentration were not statistically significant. Similar results were reported by Fryda and Visser [65] who found that samples produced by gasification contained higher content of Ca and K due to higher production temperature and lower ash content. This high content of cations could also explain less negative ζ potential of IB (−23.6 mV) when compared to AB (−26.4 mV) and NB (−27.6 mV) (Figure S5).

In spite of the different methodologies used in biochar production, there was no statistically significant difference in ζ potential between AB and NB. However, differences in surface area and pore volume were significant not only between different biochar samples, but also between samples of biochar and MSN and NC nanoparticles (Figure S6).

As evident from Figure S6, MSN had more than three times higher surface area (87.35 m^2^/g) compared to NC (25.32 m^2^/g), likely due to its mesoporous structure. The surface area of AB (59.54 m2/g) was about 1.4 times higher than NB (42.73 m^2^/g), which is in accordance with result reported by Fryda and Visser [65], demonstrating that gasification biochars tend to have higher surface area. Moreover, both AB and NB samples possessed significantly higher surface areas compared to IB (13.11 m^2^/g). Tomczyk et al. [66] also noted that higher temperature yielded biochars with higher surface area, probably due to the changes in internal structural organization. Such changes are evident in the SEM images (Figure S7).

For example, Figure S7A shows that IB sample had a more regular, sheet-like structure when compared to AB (Figure S7B) which is more irregular. The structure of NB (Figure S7C) resembles a honeycomb with highly macroporous surface. The pore volume (Figure S8) varied less in different types of biochar, but greater variation was noticed between MSN and NC. Results for pore volume in MSN and NC correlated with corresponding surface area. Similarly, pore volume was lowest in IB and the highest in NB. These findings align with Sigmund et al. [67] who reported a positive correlation between surface area and pore volume. IB had higher P/N ratio (3.74) compared to AB (0.05) and NB (1.07) (Table S4).

These results agree with Piash et al. [68] who found that increases in temperature led to an increase in P content and a decrease in N content in biochars, most likely due to increased loss of amides during production.

### 3.3. Adsorption of Bacteria

The SEM and TEM images of chitosan-coated mesoporous silica with adsorbed bacteria and nanoclay with adsorbed bacteria are shown on Figures S9A,B and S10A,B, respectively. Despite the fact that due to freezing, de-freezing, and fixing of bacteria for TEM analysis, some bacteria cells were damaged, it is evident from both Figures S9 and S10 that chitosan-coated mesoporous silica and nanoclay were adsorbed to the surface of bacteria. These results are in agreement with Jastrzębska et al. [69] for Al_2_O_3_ and Al_2_O_3_/Ag nanoparticles and Darabdhara et al. [70] for magnetic nanoparticles. From our loading studies, it was obvious that nanoclay had significantly higher loading capacity than mesoporous silica. After the final centrifugation step, the supernatant contained 32.67% non-adsorbed bacteria in case of MSN (meaning that the loaded CFU/mL was 1.35 × 10^8^). On the other hand, only 1.2% of total bacteria remained non-adsorbed to NC (meaning that the loaded CFU/mL was 1.98 × 10^8^). Given that MSN had both greater pore volume and greater surface area compared to NC (Figures S5 and S7), the likely reason for difference in loading capacity is a difference in surface charge of MSN and NC. Additionally, given that *A. vinaldii* is a Gram- negative [46] bacterium and *B. megaterium* Gram-positive [49], it is likely that their loading to positively charged surfaces will be different. However, further experiments are needed to confirm this hypothesis. Although the loading capacity of nanoclay compared to chitosan-coated mesoporous silica was significantly different, the ratio of individual bacterium adsorbed to the surface of these nanoparticles was similar. The ratio of *A. vinaldii* to *B. megaterium* adsorbed to MSN was 1.14:1, while ratio of *A. vinaldii* to *B. megaterium* adsorbed to MSN 1:1.21. The slightly higher ratio of *B. megaterium* to *A. vinaldii* adsorbed to nanoclay may be explained by the difference in charge, since Jastrzebska et al. [69] found that electrostatic interaction might be crucial during bacterial adsorption. However, differences in the shape of chitosan-coated mesoporous silica and nanoclay might also play a role. Further experiments are needed to confirm to what extent different shape and charge of bacteria and nanoparticles contribute to final distribution of bacteria on the surface of nanoparticles. SEM images of nanoparticles and biochars with adsorbed bacteria are shown in Figure S9. Interestingly, bacteria adsorbed on the surface of IB were more clustered and closer to the surface, whereas the bacteria were more uniformly adsorbed and farther from the surface the in AB and NB. Further experiments are necessary to understand how this difference in bacterial distribution impacted other examined material properties.

### 3.4. Content of P and N in Soil Substrate and Fertilizer

The P and N content in the soil substrate and fertilizer are shown in Table S5. Values for P (562.32 ± 92.96 mg/kg) and N content (229.42 ± 22.17 mg/g) were similar to those reported by Griffiths et al. [71] for a sandy loam soil. The N content in the fertilizer was similar to that reported by the manufacturer (24%), while the content of P was slightly higher than reported (30%). Additionally, both P and N content were slightly higher than the average content of conventional fertilizers [72].

### 3.5. Content of P and N in Plants’ Leaves

Table 2 and Table 3 show the P and N content of the plants’ leaves, respectively.

The biomass of the tomato plants in the 1st experiment was too low to accurately determine nutrient content. and as such the results are not shown. At the end of the experiment (after 28 days for tomato and 30 days for watermelon) and with application of 50% of the recommended dose of fertilizer, the final P content was 1.9–3.1 times lower in tomato and 2.0–2.7× lower in watermelon experiment when compared to values at start of the experiment (0 days). However, the addition of both nanoparticles with adsorbed bacteria and biochars with adsorbed bacteria increased P content (1.2–1.6 times compared to control) in plant leaves. These results are in agreement with Egamberdieva et al. [21] and Hale et al. [22]. However, the effect of individual “carriers” was different and was impacted by time of analysis. A two-way ANOVA showed that there was interaction between the type of “carrier” and the time of analysis (F = 33.34; *p* = 1.04 × 10^−77^; α < 0.05 for tomato and F = 6.49; *p* = 6.54 × 10^−10^; α < 0.05 for watermelon). The addition of MSN + B had a greater impact (P content: 3.7 ± 0.2 g/kg in tomato leaves after 28 days and 1.9 ± 0.1 g/kg in watermelon leaves after 30 days) than did NC + B addition (P content: 3.6 ± 0.5 g/kg in tomato leaves after 28 days and 1.9 ± 0.3 g/kg in watermelon leaves after 30 days). As noted above, these results could be explained by higher surface area and pore volume in MSN compared to NC, which may provide more uniform release of bacteria. The effect of biochars with added bacteria on P content increases was similar or higher than nanoparticles with loaded bacteria. The highest value was observed for NB + B (P content: 4.8 ± 0.7 g/kg in tomato leaves after 28 days and 2.1 ± 0.4 g/kg in watermelon leaves after 30 days). Although this result might seem contrary to data of Table S4 where the highest P content was present in IB, it is possible that the more uniform structure, the higher surface area and pore volume, and a significant number of macropores on the surface of NB (Figures S6, S7C and S8) could explain this effect. Conversely, IB + B and AB + B had similar impacts, which could be explained by the fact that although IB had significantly greater P content than AB (Table S3); the surface area of AB was higher and the bacteria were more homogenously distributed (Figures S6 and S7C). Additionally, samples where consortium (*A. vineladii* + *B. megaterium*) was added directly into soil also showed significantly higher P content compared to controls. These results are in agreement with by Aasfar et al. [73] and may implicate the enzymatic activity of soil as playing a significant role. Given that both *A. vineladii* and *B. megaterium* “communicate” with other rhizobial microorganisms [46,49,50,73] and stimulate both mineralization of organic material and increase in metabolic activity, it is possible that introduction of this consortium into soil increased the activity of phosphateses and phytaleses further aiding in the conversion of insoluble to soluble forms of phosphorus. However, this hypothesis needs to be further tested by measuring changes in soil enzymatic activity.

The results for N content in leaves corresponded well to the P content data. For example, NB + B was the most efficient treatment in both tomato and watermelon experiments (N content: 33.7 ± 4.3 mg/g in tomato leaves after 28 days and 24.1 ± 0.6 mg/g in watermelon leaves after 30 days). These data again would suggest that initial N content in biochars (Table S4) had less impact than the material surface characteristics and surface area. Again, in both tomato and watermelon after one month of treatment, *A. vinelandii* still exhibited a positive impact on N content. This is in agreement with Zhao et al. [74] and demonstrates that *A. vineladii* can survive the inherent enzymatic activity present in soil.

### 3.6. Chlorophyll Content

**Figure 1:**
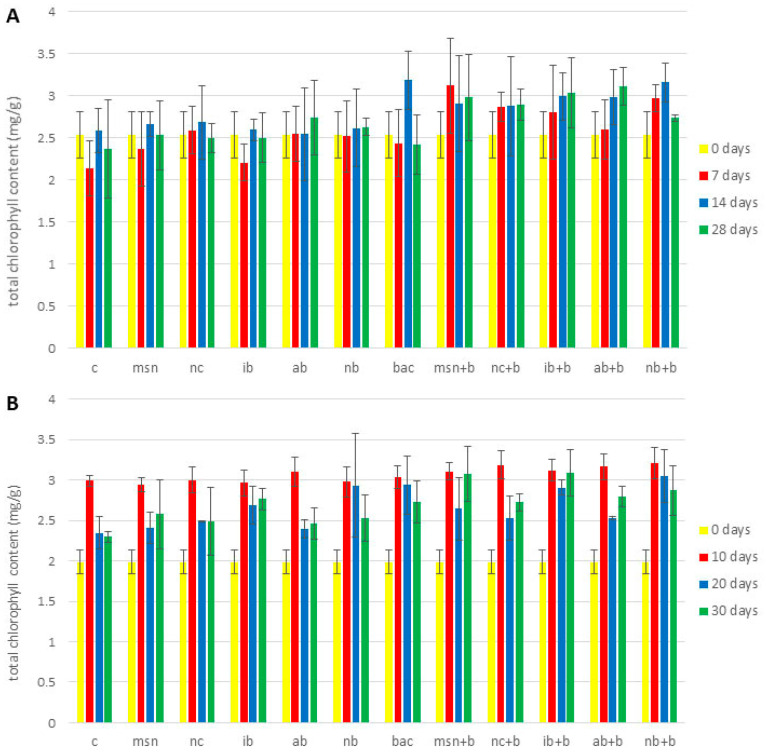
Total chlorophyll content (mg/g fresh mass). (**A**) 2nd tomato experiment; (**B**) watermelon experiment. Abbreviations: C—control; MSN—chitosan-coated mesoporous silica nanoparticles; NC—nanoclay; IB—“Italian” biochar; AB—Aries Green biochar; NB—Naked biochar; BAC—bacteria; MSN + B—chitosan-coated mesoporous silica nanoparticles with adsorbed bacteria; NC + B—nanoclay with adsorbed bacteria; IB + B—“Italian” biochar with adsorbed bacteria; AB + B—Aries Green biochar with adsorbed bacteria; NB + B—Naked biochar with adsorbed bacteria.

Figure 1 and Figure S11 show changes in total chlorophyll content during the 1st tomato experiment (Figure S11), 2nd tomato experiment (Figure 1A), and the watermelon experiment (Figure 1B). In the 1st tomato experiment (Figure S11), no significant differences in chlorophyll content were observed between treatments and the control. It is well documented that plants grown in nutrient deficient conditions show decreased photosynthesis [75,76]. Given that a lack of N will strongly impact plant metabolism [77], any differences between treatments were likely overshadowed.

Differences in chlorophyll content between control and treated plants were statistically significant at the end of both the 2nd tomato and the watermelon experiment (Figure 1). In the 2nd tomato experiment the chlorophyll content in treated plants increased 1.1–1.3 times (compared to control) after 28 days. After 30 days, chlorophyll content in treated plants increased 1.1–1.4 times compared to control. Interestingly, differences were evident only at the end of the experiments, which agrees with Hou et al. [78] who reported that the impact of biochar addition took several months to become significant. Importantly, a longer life cycle study might reveal greater differences in instances where the impact of the adsorbed bacteria was minimal after only one month.

### 3.7. Plant Cell Viability

**Figure 2:**
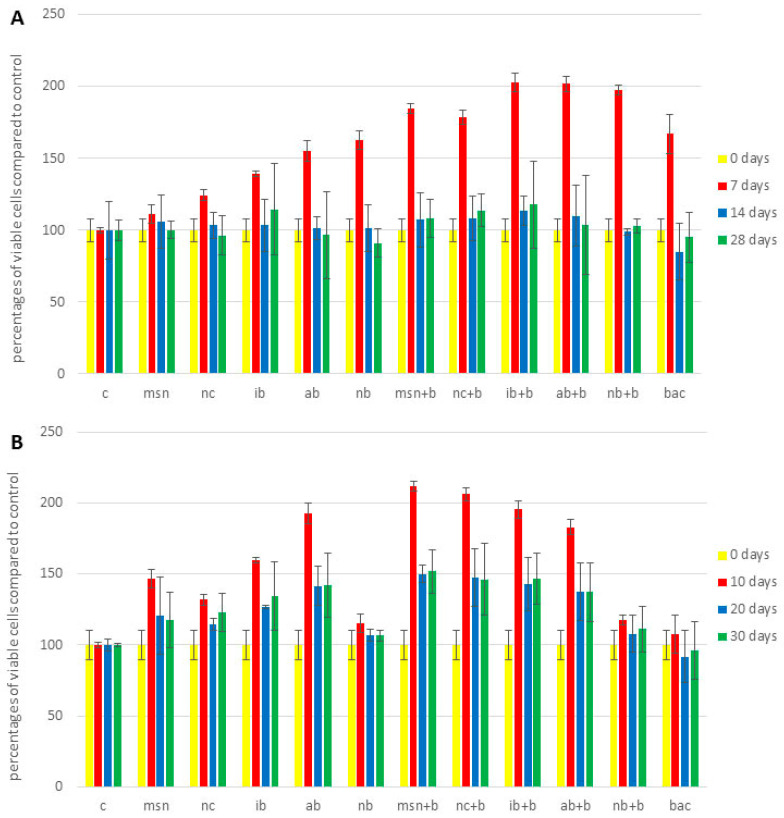
Cell viability expressed as percentages of viable cells compared to control. (**A**) 2nd tomato experiment; (**B**) watermelon experiment. Abbreviations: C—control; MSN—chitosan-coated mesoporous silica nanoparticles; NC—nanoclay; IB—“Italian” biochar; AB—Aries Green biochar; NB—Naked biochar; MSN + B—chitosan-coated mesoporous silica nanoparticles with adsorbed bacteria; NC + B—nanoclay with adsorbed bacteria; IB + B—“Italian” biochar with adsorbed bacteria; AB + B—Aries Green biochar with adsorbed bacteria; NB + B—Naked biochar with adsorbed bacteria; BAC—bacteria.

As evident in Figure 2 and Figure S12, none of the treatments exerted toxicity. In fact, compared to controls, the treatments generally increased cell viability, although no treatments were statistically significant, and perhaps more importantly the effect of individual treatments was species-dependent and influenced by the amount of fertilizer added.

In the first tomato experiment (Figure S12), the addition of NC, IB + B and NB + B had the greatest positive effect on viability. For example, after 28 days, addition of NC and IB + B increased percentage of viable cells approximately 1.2 times and 1.3 times, respectively (compared to control); while addition of NB + B increased A595 approximately 1.4 times (compared to control). In the 2nd tomato experiment (Figure 2A), IB, NC + B and IB + B had the greatest impact on viability. After 28 days, addition of IB, NC + B and IB + B increased percentage of viable cells by approximately 1.1 times, 1.2 times, and 1.3 times, respectively (compared to control). In watermelon (Figure 2B), the highest effect was observed with AB and with MSN + B and IB + B. After 30 days, addition of AB, MSN + B and IB + B increased percentage of viable cells approximately 1.4 times, 1.5 times, and 1.5 times, respectively (compared to control). As noted above, differences in results for the 1st and 2nd tomato experiment could be explained by physiological differences as a function of nutrient deficiency. Differences in the effects of individual nanoparticles can be explained by difference in zeta potential: positively charged nanoparticles such as MSN interacted more with the negatively charged cell membrane and therefore had less positive effect [79]. Additional factors that might explain different effects of MSN and NC are the shape and size of the particles. Huang et al. [80] discovered that larger and more irregular nanoparticles had more pronounced cytotoxicity. Additionally, IB had greater impact on viability compared to AB and NB. This may be explained by higher pH and higher content of K, Na, and Ca (Table S4). Cui et al. [81] found that the addition of biochar with different pH values differentially impacted plant metabolism; this is consistent with data reported in Section 3.9, where greater increases in soil pH were observed when NC and IB were applied. Additionally, a higher content of cations could have altered the activity of ion channels that mediate membrane integrity and thus influence changes in viability [82].

Interestingly, unlike results for chlorophyll, P and N content, the addition of bacteria directly in soil had no effect on cell viability in either of the experiments. Further investigation is needed to understand these findings.

### 3.8. Antioxidative Properties

**Figure 3:**
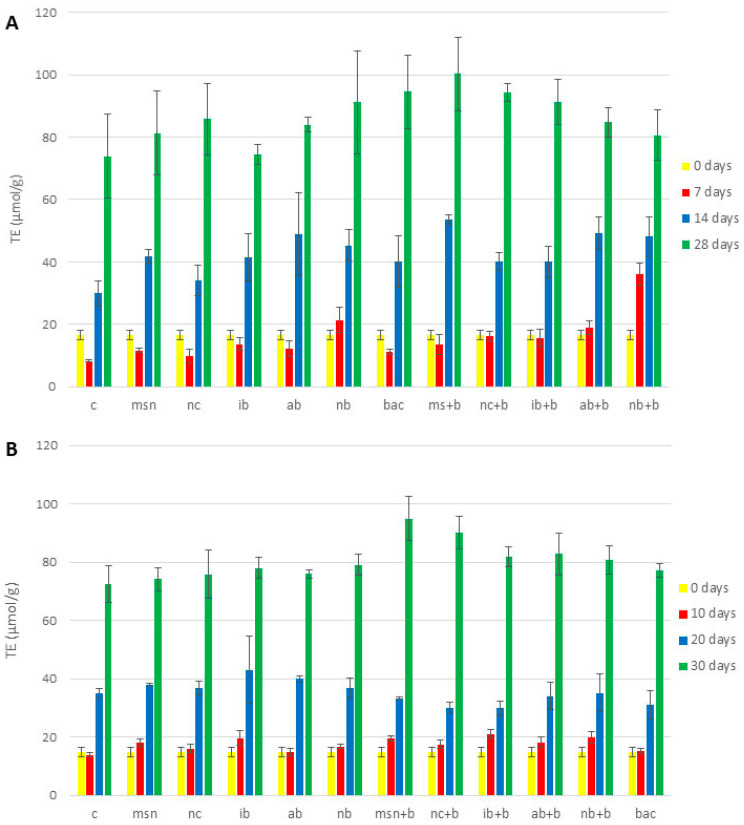
Antioxidative properties as measured by the DPPH assay. (**A**) 2nd tomato experiment; (**B**) watermelon experiment. Abbreviations: C—control; MSN—chitosan-coated mesoporous silica nanoparticles; NC—nanoclay; IB—“Italian” biochar; AB—Aries Green biochar; NB—Naked biochar; MSN + B—chitosan-coated mesoporous silica nanoparticles with adsorbed bacteria; NC + B—nanoclay with adsorbed bacteria; IB + B—“Italian” biochar with adsorbed bacteria; AB + B—Aries Green biochar with adsorbed bacteria; NB + B—Naked biochar with adsorbed bacteria; BAC—bacteria.

Given that the DPPH and ABTS assays have different sensitivity towards polar and nonpolar compounds [83], both tests were conducted to obtain a more comprehensive understanding of the antioxidative effect of the biochars and nanoparticles (Figure 3, Figures S13 and S14). As evident from Figure 3, Figures S13 and S14, the plant antioxidant capacity was increased in all 3 experiments.

These were the results of DPPH assay: In all 3 experiments, MSN + B exhibited higher antioxidative potential than NC + B. There are several possible explanations for this greater impact of MSN + B, including differences in charge, size, and porosity. Sadeghnia et al. [84] found that the same factors that contribute to increased viability also play role in the prevention of oxidative damage. Lee et al. [85] also reported that small size and spherical shape of mesoporous silica nanoparticles played a role in prevention of apoptosis and inflammation, and the authors considered the porous nature of MSN to be the key factor. Regarding the impact of biochars, in the 1st tomato experiment (Figure S13), NB + B had greater antioxidative potential than IB + B and AB + B. However, in the 2nd tomato experiment (Figure 3A) and the watermelon experiment (Figure 3B), IB + B and AB + B had the greatest impact on antioxidant potential, respectively. Hasanuzzaman et al. [86] found that addition of biochar increased the activity of several enzymes involved in antioxidative defense, including dehydroascorbate reductase, monodehydroascorbate reductase, glutathione reductase, superoxide dismutase, and catalase, among others. Additionally, Cui et al. [81] found that differences in biochar pH caused the activation of different metabolic pathways in plants. Further investigation is needed to establish the significance of pH on the impact of biochar types on different plant species.

Results of ABTS assay: Although results of ABTS assays (Figure S14) were generally similar to those of the DPPH method, there were a number of differences in the 2nd tomato experiment (Figure S14A) and watermelon experiment (Figure S14B) that were not of statistical significance. This suggests that the ABTS assay is not the best method to analyze changes in concentration of antioxidative compounds in plant leaves as a function of soil treatments.

Interestingly, in all three experiments and in both assays, the application of bacteria directly to soil had positive effects on plant antioxidant defenses. These results align well with our N and P content findings and further demonstrate the high survival rate of selected PGPR.

### 3.9. Changes in Soil pH

Table S6 shows changes in soil pH across all three experiments. As anticipated (due to the high pH of biochar samples), the addition of biochar led to increases in soil pH. However, this increase was most evident in the 1st tomato experiment. This could be explained by observations made by Hinsinger et al. [87], who reported that the composition of plant exudates from nutrient-deficient plants was different than that of plants grown under optimal conditions. This change in composition of plant exudates could readily lead to changes in soil pH. These changes could be viewed as a response to nutrient deficiency and represent an effort to obtain more nutrients from the surround media. In fact, in the 2nd tomato experiment and the watermelon experiment, the biochars had a buffering effect; the final pH value of biochar treated soil was lower or equivalent to the control. This finding is consistent with Zhang et al. [88] and may be reflective of changes in rhizobial community. However, Martisen et al. [89] reported that lower CEC values led to greater pH increases; we found no such correlation in our work. Notably, the impact of MSN and NC on soil pH was similar and comparable to that exhibited by the biochars.

### 3.10. Microbiological Analysis of Soil

**Figure 4:**
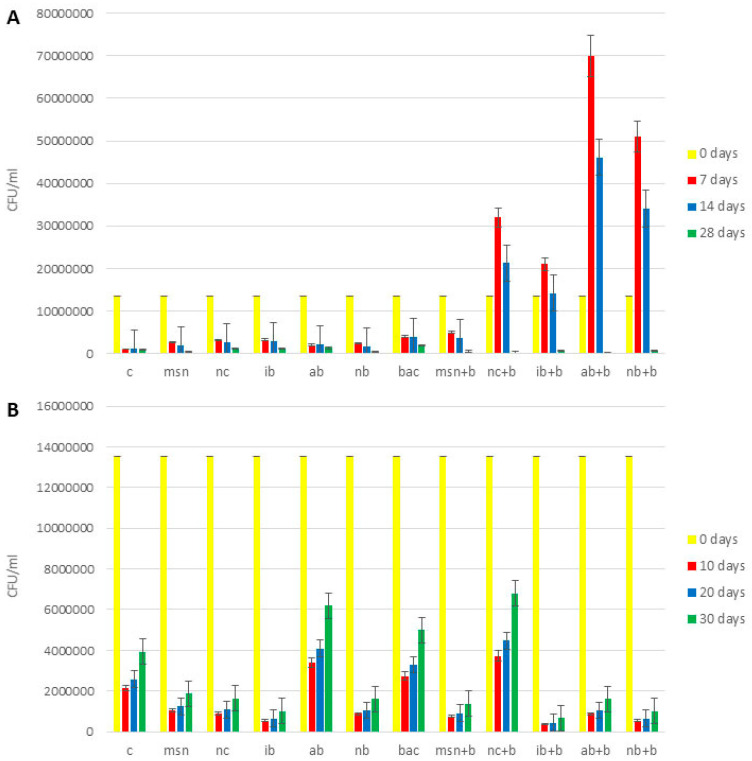
Content of total bacteria in soil. (**A**) 2nd tomato experiment; (**B**) watermelon experiment. Abbreviations: C—control; MSN—chitosan-coated mesoporous silica nanoparticles; NC—nanoclay; IB—“Italian” biochar; AB—Aries Green biochar; NB—Naked biochar; BAC—bacteria; MSN + B—chitosan-coated mesoporous silica nanoparticles with adsorbed bacteria; NC + B—nanoclay with adsorbed bacteria; IB + B—“Italian” biochar with adsorbed bacteria; AB + B—Aries Green biochar with adsorbed bacteria; NB + B—Naked biochar with adsorbed bacteria.

The content of total bacteria in all 3 experiments is shown in Figure 4 and Figure S15.

With some exceptions, across all experiments, all treatments increased bacteria compared to controls. In the 1st tomato experiment (Figure S15), the effect of NC + B on increases of total bacteria content (4.5 times higher compared to control) was greater than MSN + B (3.3 times higher compared to control) and the addition of AB + B had more positive impact than did IB + B and NB + B. The addition of AB + B increased content of total bacteria 3.8 times (compared to control), while addition of IB + B and NB + B increased content of total bacteria 2.5 times and 3.5 times, respectively (compared to control). In the 2nd tomato experiment (Figure 4A), NB + B had the greatest impact of all biochars as to adsorbed bacteria (it increased content of total bacteria 8.4 times compared to control), but MSN + B had a greater positive impact than NC + B (5.8 times and 3 times increase, respectively, compared to control). In the watermelon experiment (Figure 4B), the effect of IB + B was the highest among biochars (1.7 times increase compared to control), but NC + B had more impact that MSN + B (1.8 times increase in case of NC + B addition and 2.6 times decrease in the case of MSN + B addition, compared to control).

**Figure 5:**
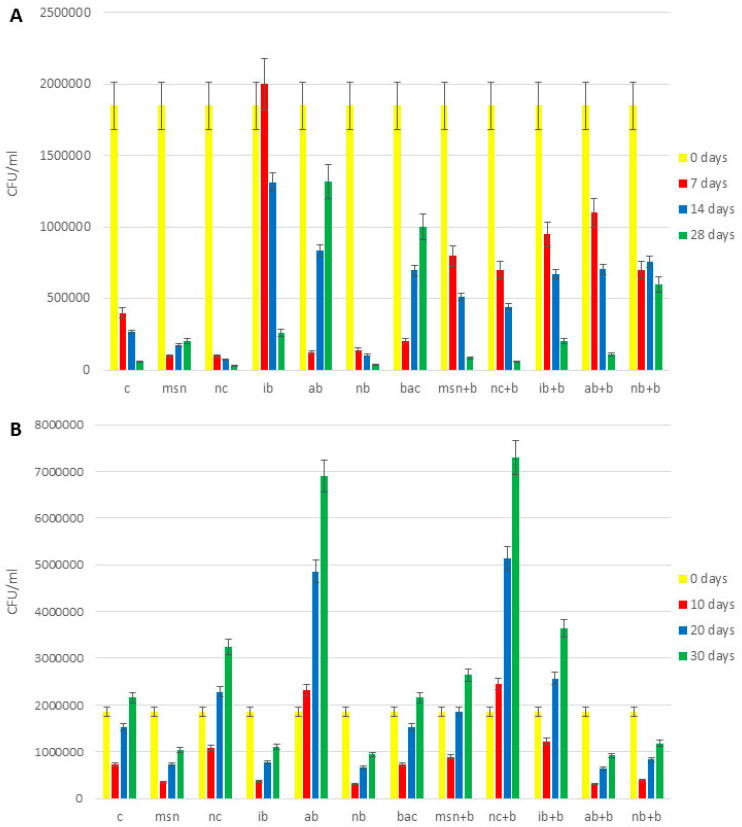
Content of nitrogen-fixing bacteria in the soil. (**A**) 2nd tomato experiment; (**B**) watermelon experiment. Abbreviations: C—control; MSN—chitosan-coated mesoporous silica nanoparticles; NC—nanoclay; IB—“Italian” biochar; AB—Aries Green biochar; NB—Naked biochar; BAC—bacteria; MSN + B—chitosan-coated mesoporous silica nanoparticles with adsorbed bacteria; NC + B—nanoclay with adsorbed bacteria; IB + B—“Italian” biochar with adsorbed bacteria; AB + B—Aries Green biochar with adsorbed bacteria; NB + B—Naked biochar with adsorbed bacteria.

The content of nitrogen-fixing bacteria in all 3 experiments in shown in Figure 5 and Figure S16. Across all experiments, all treatments increased nitrogen fixing bacteria compared to the controls. In the 1st tomato experiment, the results for the nitrogen- fixing bacteria (Figure S16) were in agreement with the findings for total bacteria. However, in the 2nd tomato experiment (Figure 5A), NB + B had the most prominent effect (10 times increase compared to control), while in watermelon experiment (Figure 5B) the highest content of nitrogen-fixing bacteria was found in soils treated with NC + B (3.4 times increase compared to control).

**Figure 6:**
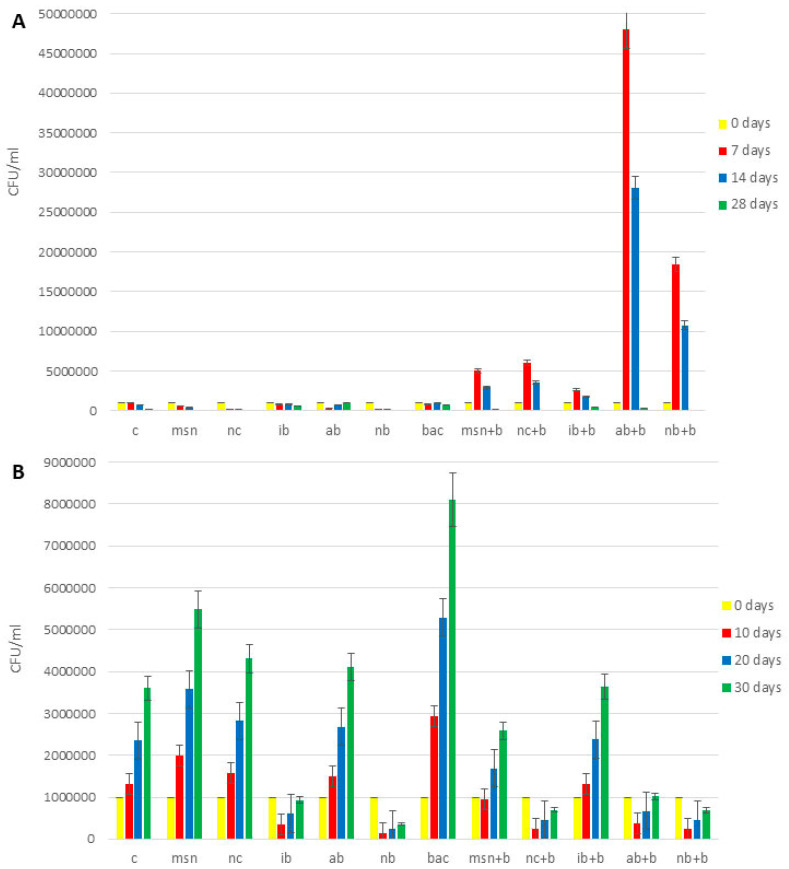
Content of phosphorus-solubilizing bacteria in the soil. (**A**) 2nd tomato experiment; (**B**) watermelon experiment. Abbreviations: C—control; MSN—chitosan-coated mesoporous silica nanoparticles; NC—nanoclay; IB—“Italian” biochar; AB—Aries Green biochar; NB—Naked biochar; BAC—bacteria; MSN + B—chitosan-coated mesoporous silica nanoparticles with adsorbed bacteria; NC + B—nanoclay with adsorbed bacteria; IB + B—“Italian” biochar with adsorbed bacteria; AB + B—Aries Green biochar with adsorbed bacteria; NB + B—Naked biochar with adsorbed bacteria.

The content of phosphorus-solubilizing bacteria is shown in Figure 6 and Figure S17. Similar to the data above, all treatments increased bacterial numbers compared to controls. Results for the 1st tomato experiment (Figure S17), 2nd tomato experiment (Figure 6A), and the watermelon experiment (Figure 6B) are similar to those reported for nitrogen-fixing bacteria.

These results are in accordance with Husna et al. [16] and Hansen et al. [23], both of whom found that the addition of biochar increased the content of beneficial bacteria in soil, and also with Yang et al. [24], who demonstrated that the type of biochar strongly influenced the rhizobial community, by causing differential increase in PGPR content. Also, in all experiments, the addition of bacteria directly to the soil led to increases in phosphorus-solubilizing and nitrogen-fixing bacteria (compared to control), which confirmed that bacteria were not degraded by soil enzymatic activity.

### 3.11. Plants Biomass

The results for total plant biomass, stem length and root mass in the 2nd tomato experiment and watermelon experiment are shown in Table 4. Although only 50% of the recommended dose of fertilizer was used, plant growth was robust, which suggests that addition of nanoparticles and/or biochar and bacteria provided sufficient P and N.

There were no significant differences in root mass as a function of treatment in either the tomato or watermelon experiments. The reason for this lack of effect is not known but we do note that the root mass is quite low in general, and it is possible that small differences as a function of treatment are therefore not evident. However, in both experiments there were statistically significant differences in total mass and stem length. In the tomato experiments, a significant increase in total mass was observed with MSN + B and IB + B (1.2 times and 1.1 times increase compared to control, respectively), while significant differences in stem length were observed for samples MSN, NC, MSN + B, NC + B, IB + B and AB + B (1.1–1.2 times increase compared to control). Taken together, these results demonstrate that tomato responded best to the treatments of bacteria with chitosan-coated mesoporous silica and bacteria with the “Italian” biochar. These data align well with the results from the antioxidant properties and viability assay, and further demonstrate the benefit of the carrier on the plant. Differences in stem length that did not agree with total plant mass, such as MSN, NC, NC + B, and AB + B, could reflect increased rates of nutrient transport through the plant. For example, in the watermelon experiment, differences in total mass and stem length were more pronounced than in the tomato experiment. In this experiment, all samples with adsorbed bacteria (MSN + B, NC + B, IB + B, AB + B and NB + B) showed significant increases when compared to controls (1.1–1.3 times increase in total mass and 1.2–1.5 times increase in stem length). These results demonstrate that watermelon was more sensitive to the presence of bacteria and less sensitive to the type of “carrier” when compared to tomato. All treated plants showed increases in stem length when compared to controls, with the greatest differences observed for samples NC, AB, NC + B and AB + B. It is evident that that although both types of plant responded positively to the presence of bacteria, response to the type of carrier was different, with tomato being more impacted by MSN and IB and watermelon being more impacted by NC and AB. Additionally, a two-way ANOVA also showed that in watermelon there was an interaction between whole plant mass and stem length (F = 5942.74; *p* = 1.17 × 10^−80^; α < 0.05); this is not evident for tomato. This may be a function of the length of the plant life cycles: tomato generally requires approximately 80 days until fruit formation, depending on cultivar, whereas watermelon requires approximately 60 days, depending on cultivar [90,91]. Thus, it is possible that such interactions would be observed for tomato as well if adjusted for different lengths of growth cycle. Further investigation is needed to establish the cause of this interaction and why it was not observed for both plant species.

## 4. Conclusions

The use of biochars and nanoparticles as carriers for PGPR led to several positive outcomes on watermelon and tomato growth, including increases in the content of P and N, chlorophyll, viability, antioxidative potential, and total plant mass, as well as increases in nitrogen-fixing and phosphorus-solubilizing bacteria. Importantly, the magnitude of benefit for the individual types of nanoparticles and biochar was plant species dependent, as well as being impacted by overall nutrient status. Further experiments are needed to establish connection between type of plant and treatment. This work adds to our understanding of: (1) the effect of biochar produced by different methodologies on plants’ growth, viability, immunity, and nutrient content; (2) the different impacts of chitosan-coated mesoporous silica nanoparticles and nanoclay on plants’ growth, viability, immunity, and nutrient content; and (3) the potential to increase effect of PGPR by using biochar and nanoparticles as carriers.

## Figures and Tables

**Table 1:** Treatments used in experiments.

	Control (C)	
	Treatment with Bacteria (BAC)	
chitosan-coated mesoporous silica	treatment with chitosan-coated mesoporous silica (MSN)	treatment with chitosan- coated mesoporous silica with loaded bacteria (MSN + B)
nanoclay	treatment with nanoclay (NC)	treatment with nanoclay with loaded bacteria (NC + B)
“Italian” biochar	treatment with “Italian” biochar (IB)	treatment with “Italian” biochar with loaded bacteria (IB + B)
Aries biochar	treatment with Aries green biochar (AB)	treatment with Aries Green biochar with loaded bacteria (AB + B)
Naked biochar	treatment with Naked biochar (NB),	treatment with Naked biochar with loaded bacteria (NB + B).

**Table 2:** P content (mg/g) in the 2nd tomato experiment and watermelon experiment *.

Sample	Tomato Experiment P Content (mg/g)				Watermelon Experiment P Content (mg/g)			
	0 Days	7 Days	14 Days	28 Days	0 Days	10 Days	20 Days	30 Days
C	9.3 ± 0.5 ^a^	3.6 ± 0.4 ^a^	3.5 ± 0.2 ^a^	3.0 ± 0.4 ^a^	4.3 ± 0.6 ^a^	4.7 ± 0.5 ^a^	1.9 ± 0.3 ^a^	1.6 ± 0.2 ^a^
MSN	9.3 ± 0.5 ^a^	3.7 ± 0.3 ^a^	3.9 ± 0.1 ^b^	3.3 ± 0.3 ^a^	4.3 ± 0.6 ^a^	3.2 ± 0.4 ^b^	1.9 ± 0.1 ^a^	1.8 ± 0.3 ^a^
NC	9.3 ± 0.5 ^a^	3.6 ± 0.3 ^a^	4.7 ± 0.2 ^c^	3.3 ± 0.5 ^a^	4.3 ± 0.6 ^a^	2.9 ± 0.5 ^b^	2.4 ± 0.5 ^b^	1.6 ± 0.2 ^a^
IB	9.3 ± 0.5 ^a^	3.7 ± 0.2 ^a^	4.3 ± 0.4 ^b,c^	3.4 ± 0.4 ^a^	4.3 ± 0.6 ^a^	3.7 ± 0.4 ^c^	2.9 ± 0.6 ^b^	2.1 ± 0.3 ^b^
AB	9.3 ± 0.5 ^a^	3.4 ± 0.4 ^a^	5.0 ± 0.5 ^c^	3.5 ± 0.3 ^a,b^	4.3 ± 0.6 ^a^	3.5 ± 0.^b,c^	2.6 ± 0.5 ^b^	2.0 ± 0.2 ^b^
NB	9.3 ± 0.5 ^a^	3.2 ± 0.6 ^a^	4.7 ± 0.3 ^c^	3.9 ± 0.4 ^b^	4.3 ± 0.6 ^a^	3.6 ± 0.5 ^b,c^	2.4 ± 0.1 ^b^	1.8 ± 0.3 ^a^
BAC	9.3 ± 0.5 ^a^	5.0 ± 0.4 ^b^	6.5 ± 0.7 ^d^	4.3 ± 0.4 ^b,c^	4.3 ± 0.6 ^a^	3.8 ± 0.4 ^c^	2.8 ± 0.4 ^b^	2.3 ± 0.4 ^b^
MSN + B	9.3 ± 0.5 ^a^	4.6 ± 0.3 ^a,b^	4.3 ± 0.2 ^b,c^	3.7 ± 0.2 ^b^	4.3 ± 0.6 ^a^	2.9 ± 0.5 ^b^	2.0 ± 0.6 ^a^	1.9 ± 0.1 ^b^
NC + B	9.3 ± 0.5 ^a^	4.8 ± 0.3 ^a,b^	4.5 ± 0.5 ^b,c^	3.6 ± 0.5 ^a,b^	4.3 ± 0.6 ^a^	3.2 ± 0.2 ^b^	2.4 ± 0.4 ^b^	1.9 ± 0.3 ^a,b^
IB + B	9.3 ± 0.5 ^a^	4.0 ± 0.4 ^a^	5.2 ± 0.8 ^c^	3.9 ± 0.3 ^b^	4.3 ± 0.6 ^a^	3.6 ± 0.8 ^b,c^	2.5 ± 0.6 ^b^	1.9 ± 0.1 ^b^
AB + B	9.3 ± 0.5 ^a^	4.3 ± 0.4 ^a^	4.7 ± 0.6 ^b^	3.8 ± 0.4 ^a,b^	4.3 ± 0.6 ^a^	3.9 ± 0.8 ^c^	2.1 ± 0.3 ^a^	2.0 ± 0.1 ^b^
NB + B	9.3 ± 0.5 ^a^	3.8 ± 0.5 ^a^	5.0 ± 0.6 ^b,c^	4.8 ± 0.7 ^b,c^	4.3 ± 0.6 ^a^	3.6 ± 0.2 ^b,c^	2.8 ± 0.5 ^b^	2.1 ± 0.4 ^b^

* Different letters at the same column means that differences between samples were statistically significant (determined by Tukey test). Abbreviations: C—control; MSN—chitosan-coated mesoporous silica nanoparticles; NC—nanoclay; IB—“Italian” biochar; AB—Aries Green biochar; NB—Naked biochar; BAC—bacteria; MSN + B—chitosan-coated mesoporous silica nanoparticles with adsorbed bacteria; NC + B—nanoclay with adsorbed bacteria; IB + B—“Italian” biochar with adsorbed bacteria; AB + B—Aries Green biochar with adsorbed bacteria; NB + B—Naked biochar with adsorbed bacteria.

**Table 3:** N content (mg/g) in the 2nd tomato experiment and watermelon experiment *.

Sample	Tomato Experiment N Content (mg/g)				Watermelon Experiment N Content (mg/g)			
	0 Days	7 Days	14 Days	28 Days	0 Days	10 Days	20 Days	30 Days
C	57.1 ± 1.2 ^a^	54.8 ± 2.0 ^a^	41.4 ± 2.3 ^a^	22.0 ± 3.2 ^a^	29.9 ± 3.1 ^a^	25.4 ± 1.4 ^a^	18.5± 0.3 ^a^	17.7 ± 1.5 ^a^
MSN	57.1 ± 1.2 ^a^	44.7 ± 1.7 ^b^	39.1 ± 1.3 ^a^	22.9 ± 1.8 ^a^	29.9 ± 3.1 ^a^	28.2 ± 4.6 ^a^	19.8 ± 1.1 ^a^	20.0 ± 2.4 ^a^
NC	57.1 ± 1.2 ^a^	42.6 ± 0.8 ^c^	38.8 ± 2.8 ^a^	23.4 ± 2.6 ^a^	29.9 ± 3.1 ^a^	24.9 ± 2.5 ^a^	20.2 ± 1.6 ^a^	21.9 ± 2.6 ^a^
IB	57.1 ± 1.2 ^a^	46.0 ± 4.3 ^b^	39.7 ± 2.5 ^a^	22.2 ± 3.1 ^a^	29.9 ± 3.1 ^a^	27.2 ± 2.1 ^a^	20.8 ± 0.4 ^a^	19.0 ± 2.4 ^a^
AB	57.1 ± 1.2 ^a^	51.7 ± 0.9 ^d^	36.5 ± 2.2 ^b^	22.3 ± 3.6 ^a^	29.9 ± 3.1 ^a^	24.8 ± 3.0 ^a^	18.8 ± 0.3 ^a^	18.7 ± 1.6 ^a^
NB	57.1 ± 1.2 ^a^	55.8 ± 4.6 ^a^	34.9 ± 3.1 ^b^	28.8 ± 3.3 ^b^	29.9 ± 3.1 ^a^	28.5 ± 0.7 ^a^	20.2 ± 0.4 ^a^	19.3 ± 2.2 ^a^
BAC	57.1 ± 1.2 ^a^	53.5 ± 4.1 ^a^	44.5 ± 2.4 ^a^	25.2 ± 2.4 ^c^	29.9 ± 3.1 ^a^	25.6 ± 2.9 ^a^	25.4 ± 2.9 ^b^	21.6 ± 0.8 ^c^
MSN + B	57.1 ± 1.2 ^a^	47.4 ± 3.1 ^b^	38.4 ± 2.3 ^a^	24.1 ± 2.2 ^c^	29.9 ± 3.1 ^a^	27.5 ± 3.4 ^a^	20.7 ± 3.4 ^a^	22.2 ± 2.4 ^b,c^
NC + B	57.1 ± 1.2 ^a^	48.3 ± 1.2 ^d^	38.9 ± 2.2 ^a^	22.2 ± 1.1 ^a^	29.9 ± 3.1 ^a^	23.2 ± 3.0 ^a^	18.3 ± 3.0 ^a^	21.6 ± 2.6 ^b^
IB + B	57.1 ± 1.2 ^a^	44.4 ± 4.1 ^b,c^	39.0± 1.5 ^a^	28.3 ± 1.0 ^c,d^	29.9 ± 3.1 ^a^	27.8 ± 2.2 ^a^	19.9 ± 2.2 ^a^	20.9 ± 1.8 ^b,c^
AB + B	57.1 ± 1.2 ^a^	51.7 ± 2.4 ^d^	38.9 ± 3.2 ^a^	32.2 ± 3.7 ^d,e^	29.9 ± 3.1 ^a^	29.9 ± 3.9 ^a^	19.5 ± 3.9 ^a^	21.6 ± 2.8 ^b^
NB + B	57.1 ± 1.2 ^a^	53.2 ± 4.7 ^a^	41.5 ± 2.0 ^a^	33.7 ± 4.3 ^d,e^	29.9 ± 3.1 ^a^	28.7 ± 3.3 ^a^	20.7 ± 3.3 ^a^	24.1 ± 0.6 ^c^

* Different letters at the same column means that differences between samples were statistically significant (determined by Tukey test). Abbreviations: C—control; MSN—chitosan-coated mesoporous silica nanoparticles; NC—nanoclay; IB—“Italian” biochar; AB—Aries Green biochar; NB—Naked biochar; BAC—bacteria; MSN + B—chitosan-coated mesoporous silica nanoparticles with adsorbed bacteria; NC + B—nanoclay with adsorbed bacteria; IB + B—“Italian” biochar with adsorbed bacteria; AB + B—Aries Green biochar with adsorbed bacteria; NB + B—Naked biochar with adsorbed bacteria.

**Table 4:** Biomass measurements in 2nd tomato and watermelon experiment.

Sample	Tomato Experiment			Watermelon Experiment		
	Total Mass (g)	Root Mass (g)	Stem Length (cm)	Total Mass (g)	Root Mass (g)	Stem Length (cm)
C	18.35 ± 2.09 ^a^	5.97 ±1.14 ^a^	23.66 ± 2.31 ^a^	10.69 ± 2.16 ^a^	1.25 ± 0.29 ^a^	37.97 ± 3.61 ^a^
MSN	19.15 ± 2.74 ^a^	6.48 ± 1.25 ^a^	26.75 ± 1.26 ^b^	10.78 ± 0.63 ^a^	1.09 ± 0.22 ^a^	43.65 ± 4.97 ^b^
NC	18.37 ± 2.41 ^a^	6.51 ± 0.84 ^a^	26.32 ± 2.77 ^b^	11.36 ± 2.11 ^a^	0.97 ± 0.11 ^a^	53.16 ± 5.43 ^c^
IB	18.69 ± 2.63 ^a^	5.99 ± 0.68 ^a^	25.01 ± 2.49 ^a^	10.82 ± 0.95 ^a^	1.04 ± 0.14 ^a^	45.46 ± 3.48 ^b^
AB	19.05 ± 2.51 ^a^	6.08 ± 0.76 ^a^	25.61 ± 2.19 ^a^	11.22 ± 0.91 ^a^	0.95 ± 0.12 ^a^	52.30 ± 6.14 ^c^
NB	18.46 ± 1.95 ^a^	6.35 ± 1.16 ^a^	24.84 ± 3.10 ^a^	11.86 ± 1.66 ^a^	1.03 ± 0.20 ^a^	47.30 ± 4.05 ^b^
BAC	18.19 ± 1.76 ^a^	5.91 ± 0.56 ^a^	22.86 ± 2.36 ^a^	11.02 ± 1.26 ^a^	0.75 ± 0.16 ^a^	48.35 ± 4.41 ^d^
MSN + B	21.99 ± 1.51 ^b^	5.99 ± 1.13 ^a^	27.54 ± 2.31 ^c^	11.89 ± 0.59 ^b^	0.92 ± 0.19 ^a^	45.22 ± 4.26 ^b^
NC + B	19.65 ± 1.72 ^a^	5.62 ± 0.70 ^a^	27.50 ± 2.24 ^c^	12.65 ± 2.02 ^b^	0.89 ± 0.17 ^a^	55.45 ± 3.02 ^c^
IB + B	20.52 ± 2.55 ^b^	6.53 ± 0.66 ^a^	25.58 ± 1.49 ^b^	12.11 ± 1.09 ^b^	0.74 ± 0.11 ^a^	50.48 ± 4.74 ^d^
AB + B	19.87 ± 1.07 ^a^	5.81 ± 0.67 ^a^	26.90 ± 3.19 ^b^	13.68 ± 1.61 ^b^	0.94 ± 0.18 ^a^	56.64 ± 4.01 ^c^
NB + B	18.93 ± 1.84 ^a^	5.93 ± 1.21 ^a^	23.81 ± 1.98 ^a^	12.28 ± 1.89 ^b^	0.72 ± 0.08 ^a^	50.18 ± 4.23 ^d^

Different letters at the same column means that differences between samples were statistically significant (determined by Tukey test). Abbreviations: C—control; MSN—chitosan-coated mesoporous silica nanoparticles; NC—nanoclay; IB—“Italian” biochar; AB—Aries Green biochar; NB—Naked biochar; BAC—bacteria; MSN + B—chitosan-coated mesoporous silica nanoparticles with adsorbed bacteria; NC + B—nanoclay with adsorbed bacteria; IB + B—“Italian” biochar with adsorbed bacteria; AB + B—Aries Green biochar with adsorbed bacteria; NB + B—Naked biochar with adsorbed bacteria.

## Data Availability

The data is available on reasonable request from the corresponding author.

## Supplementary Materials

The following supporting information can be downloaded at: https://www.mdpi.com/article/10.3390/nano12244474/s1, Figure S1: Representative transmission electron microscopy (TEM) image of chitosan-coated mesoporous silica; Figure S2: Representative transmission electron microscopy (TEM) image of nanoclay; Figure S3: Energy dispersive X-ray spectroscopy (EDX) mapping of nanoclay. Figure S4: Content of potassium (K), calcium (Ca) and sodium (Na) in biochar; Abbreviations: IB—“Italian” biochar; AB—Aries Green biochar; NB-Naked biochar, Figure S5: ζ potential of biochars. Abbreviations: IB—“Italian” biochar; AB—Aries Green biochar; NB-Naked biochar; Figure S6: Surface area of nanoparticles and biochars. Abbreviations: MSN—chitosan coated mesoporous silica; NC—nanoclay; IB—“Italian” biochar; AB—Aries Green biochar; NB-Naked biochar, Figure S7: Representative scanning electron microscopy (SEM) images of biochars. A—“Italian” biochar; B—Aries Green biochar; C-Naked biochar; Figure S8: Pore volume of nanoparticles and biochars. Abbreviations: S1—chitosan coated mesoporous silica; S2—nanoclay; S3—“Italian” biochar; S4—Aries Green biochar; S5—Naked biochar; Figure S9: Representative scanning electron microscopy (SEM) images of nanoparticles and biochars with adsorbed bacteria. A—chitosan-coated mesoporous silica + bacteria; B—nanoclay + bacteria; C—“Italian” biochar + bacteria; D—Aries Green biochar + bacteria; E—Naked biochar + bacteria; Figure S10: Representative transmission electron microscopy (TEM) images of nanoparticles with adsorbed bacteria. A—chitosan-coated mesoporous silica + bacteria; B—nanoclay + bacteria; Figure S11: Total chlorophyll content (mg/g fresh mass) in 1st tomato experiment. Abbreviations: C—control; MSN—chitosan-coated mesoporous silica nanoparticles; NC—nanoclay; IB—“Italian” biochar; AB—Aries Green biochar; NB—Naked biochar; B—bacteria; MSN + B—chitosan-coated mesoporous silica nanoparticles with adsorbed bacteria; NC + B—nanoclay with adsorbed bacteria; IB + B—“Italian” biochar with adsorbed bacteria; AB + B—Aries Green biochar with adsorbed bacteria; NB + B—Naked biochar with adsorbed bacteria; Figure S12: Cell viability in 1st tomato experiment. Abbreviations: C—control; MSN—chitosan-coated mesoporous silica nanoparticles; NC—nanoclay; IB—“Italian” biochar; AB—Aries Green bio-char; NB—Naked biochar; B—bacteria; MSN + B—chitosan-coated mesoporous silica nanoparticles with adsorbed bacteria; NC + B—nanoclay with adsorbed bacteria; IB + B—“Italian” biochar with adsorbed bacteria; AB + B—Aries Green biochar with adsorbed bacteria; NB + B—Naked biochar with adsorbed bacteria; Figure S13: Antioxidative properties in thein 1st tomato experiment measured by DPPH. Abbreviations: C—control; MSN—chitosan-coated mesoporous silica nanoparticles; NC—nanoclay; IB—“Italian” biochar; AB—Aries Green bio-char; NB—Naked biochar; B—bacteria; MSN + B—chitosan-coated mesoporous silica nanoparticles with adsorbed bacteria; NC + B—nanoclay with adsorbed bacteria; IB + B—“Italian” biochar with adsorbed bacteria; AB + B—Aries Green biochar with adsorbed bacteria; NB + B—Naked biochar with adsorbed bacteria; Figure S14:Antioxidative properties measured by ABTS assay. A—1st tomato experiment; B—2nd tomato experiment; C—watermelon experiment. Abbreviations: C—control; MSN—chitosan-coated mesoporous silica nanoparticles; NC—nanoclay; IB—“Italian” biochar; AB—Aries Green biochar; NB—Naked biochar; BAC—bacteria; MSN + B—chitosan-coated mesoporous silica nanoparticles with adsorbed bacteria; NC + B—nanoclay with adsorbed bacteria; IB + B—“Italian” biochar with adsorbed bacteria; AB + B—Aries Green biochar with adsorbed bacteria; NB + B—Naked biochar with adsorbed bacteria; Figure S15: Content of total bacteria in the soil in 1st tomato experiment. Abbreviations: C—control; MSN—chitosan-coated mesoporous silica nanoparticles; NC—nanoclay; IB—“Italian” biochar; AB—Aries Green bio-char; NB—Naked biochar; B—bacteria; MSN + B—chitosan-coated mesoporous silica nanoparticles with adsorbed bacteria; NC + B—nanoclay with adsorbed bacteria; IB + B—“Italian” biochar with adsorbed bacteria; AB + B—Aries Green biochar with adsorbed bacteria; NB + B—Naked biochar with adsorbed bacteria; Figure S16: Content of nitrogen-fixing bacteria in the soil in 1st tomato experiment. Abbreviations: C—control; MSN—chi-tosan-coated mesoporous silica nanoparticles; NC—nanoclay; IB—“Italian” biochar; AB—Aries Green bio-char; NB—Naked biochar; B—bacteria; MSN + B—chitosan-coated mesoporous silica nanoparticles with adsorbed bacteria; NC + B—nanoclay with adsorbed bacteria; IB + B—“Italian” biochar with adsorbed bacteria; AB + B—Aries Green biochar with adsorbed bacteria; NB + B—Naked biochar with adsorbed bacteria; Figure S17: Content of phosphorus-solubilizing bacteria in 1st tomato experiment. Abbreviations: C—control; MSN—chitosan-coated mesoporous silica nanoparticles; NC—nanoclay; IB—“Italian” biochar; AB—Aries Green biochar; NB—Naked biochar; B—bacteria; MSN + B—chitosan-coated mesoporous silica nanoparticles with adsorbed bacteria; NC + B—nanoclay with adsorbed bacteria; IB + B—“Italian” biochar with adsorbed bacteria; AB + B—Aries Green biochar with adsorbed bacteria; NB + B—Naked biochar with adsorbed bacteria; Table S1: Elemental analysis of bentonite nanoclay by by inductively coupled plasma optical emission spectrophotometry (ICP-OES); Table S2: Elemental analysis of bentonite nanoclay by energy dispersive X-ray spectroscopy (EDX); Table S3: pH, conductivity and cation exchange capacity (CEC) of biochar; Table S4: Content of phosphorus (P) and nitrogen (N) in biochars; Table S5: Content of P and N in soil substrate and fertilizer; Table S6: Changes in soil pH during the 1st tomato experiment, 2nd tomato experiment and watermelon experiment.
