# Supplementary material for: Engineered Nanoparticles, Natural Nanoclay and Biochar, as Carriers of Plant-Growth Promoting Bacteria
Source: Nanomaterials (Basel). 2022 Dec 17;12(24):4474. doi: 10.3390/nano12244474 (PMC9783841; doi:10.3390/nano12244474)
Supplement: Supplementary file 1 [file nanomaterials-12-04474-s001.zip › nanomaterials-2004778-supplementary.pdf]

Supplementary Materials

Engineered Nanoparticles, Natural Nanoclay and Biochar, as Carriers of Plant-Growth Promoting Bacteria

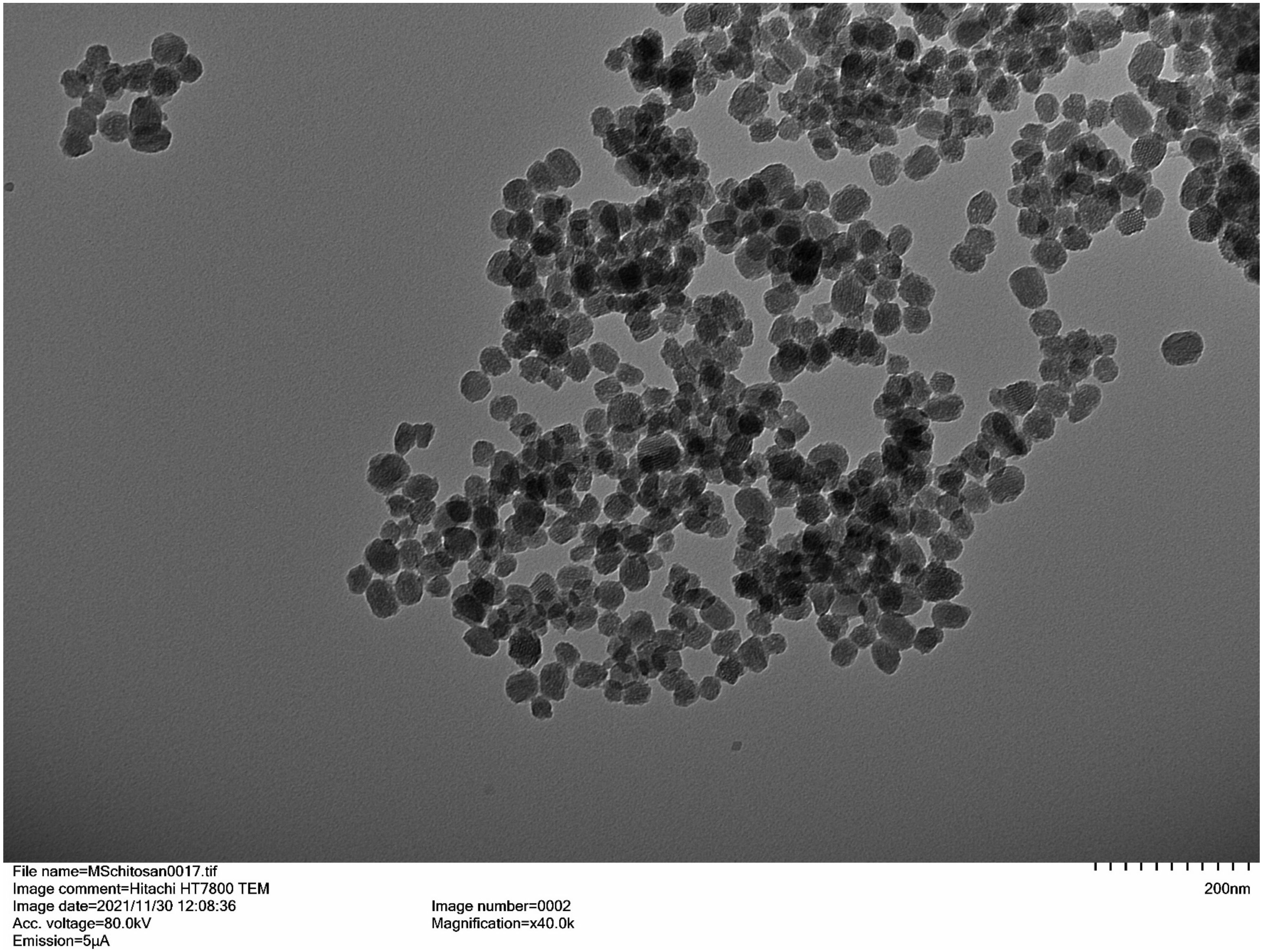

Figure S1: Representative transmission electron microscopy (TEM) image of chitosan-coated mesoporous silica.

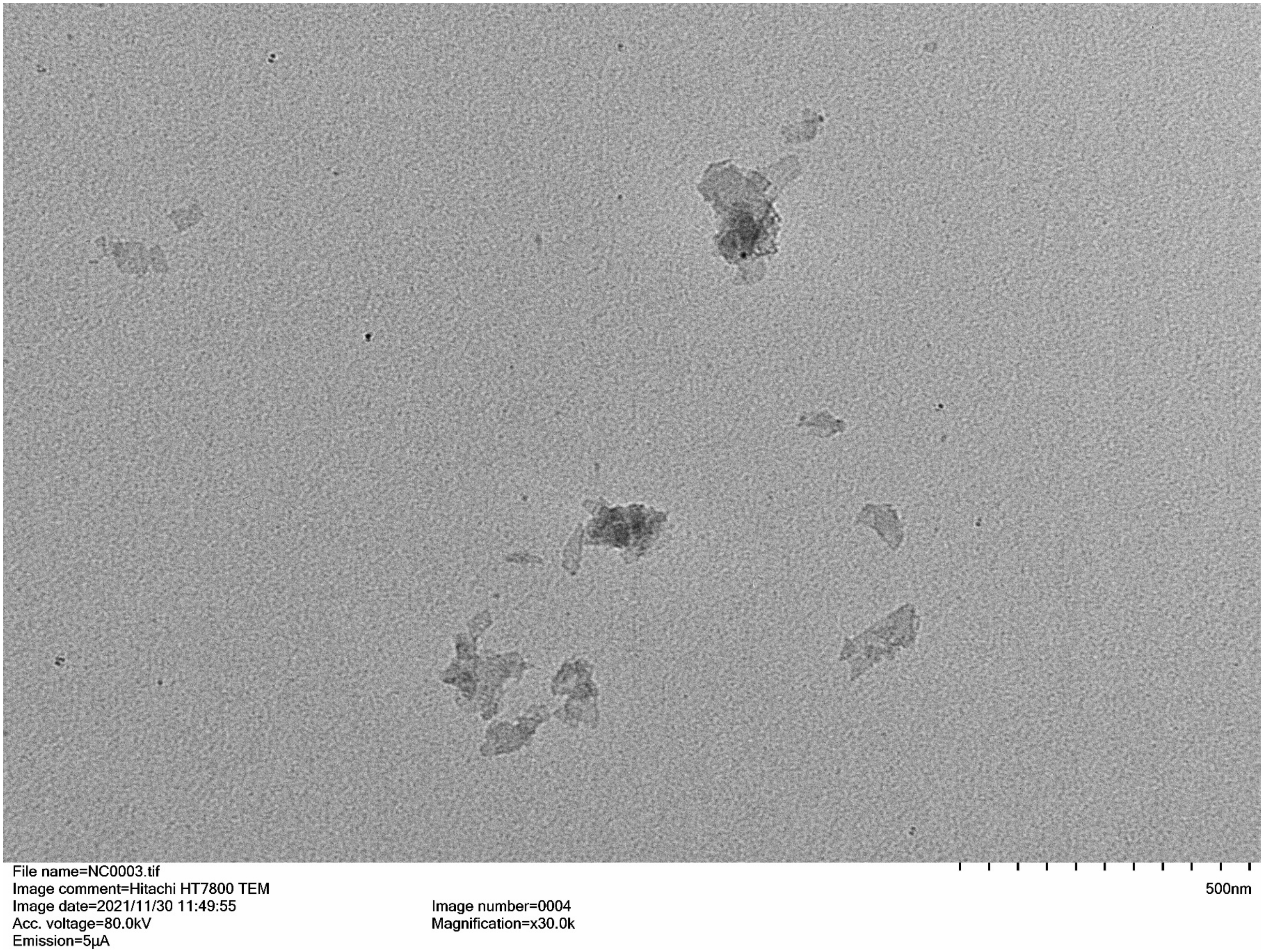

Figure S2: Representative transmission electron microscopy (TEM) image of nanoclay.

Table S1: Elemental analysis of bentonite nanoclay by inductively coupled plasma optical emission spectrophotometry (ICP-OES).

	Elements (g/kg)						
	O	Si	Al	Na	Ca	Mg	Fe
sample	452.62 ± 27.83	330.81±17.60	161.45±10.98	29.61±2.05	12.93±0.76	10.81±0.96	1.72±0.02

Table S2: Elemental analysis of bentonite nanoclay by energy dispersive X-ray spectroscopy (EDX).

Map Sum Spectrum		
Element	Weight %	σ
Ni	39.3	0.1
C	37.6	0.1
Cu	10.7	0.0
O	5.2	0.0
Si	2.8	0.0
Au	1.1	0.0
Al	1.1	0.0
Fe	1.0	0.0
Co	0.9	0.0
Mg	0.3	0.0
K	0.1	0.0
N	0.0	0.0
P	0.0	0.0
Zn	0.0	0.0

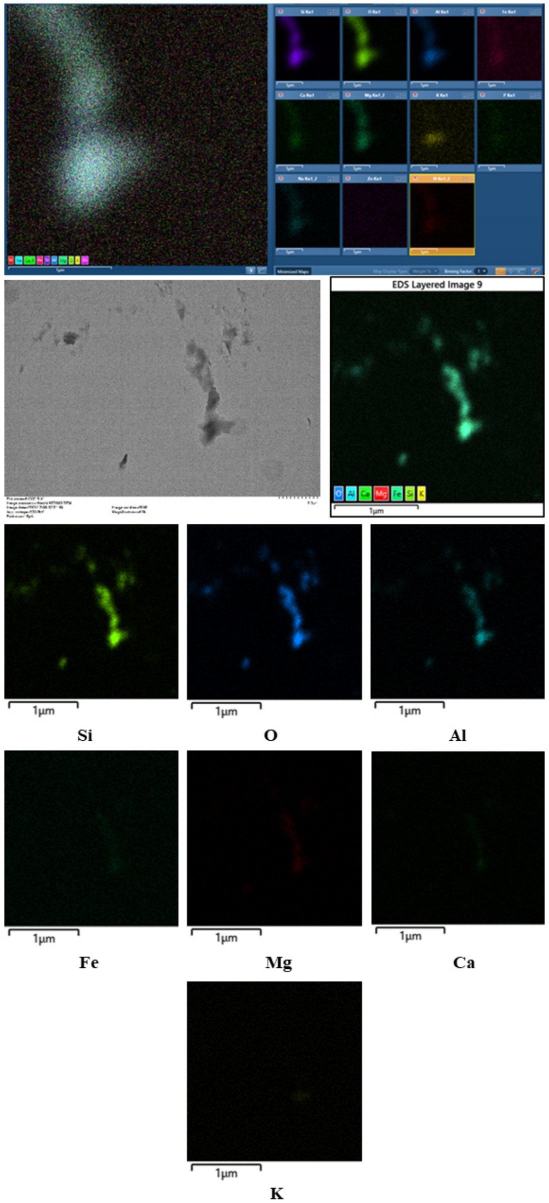

Figure S3: Energy dispersive X-ray spectroscopy (EDX) mapping of nanoclay.

Table S3: pH, conductivity and cation exchange capacity (CEC) of biochars

sample	pH	Electric conductivity (μS/cm)	CEC (cmol/kg)
Aries Green biochar (AB)	9.29 ± 0.06[a]	459 ± 2.1[a]	34.7 ± 2.9[a]
Naked biochar (NB)	9.51 ± 0.08[a]	767 ± 3.4[b]	24.5 ± 1.3[b]
"Italian" biochar (IB)	10.62 ± 0.03[b]	898 ± 4.2[c]	39.1 ± 2.8[a]

*Abbreviation: CEC- cation exchange capacity; different letters in the same column means that differences between samples were statistically significant (determined by Tukey test at $p < 0.05$)

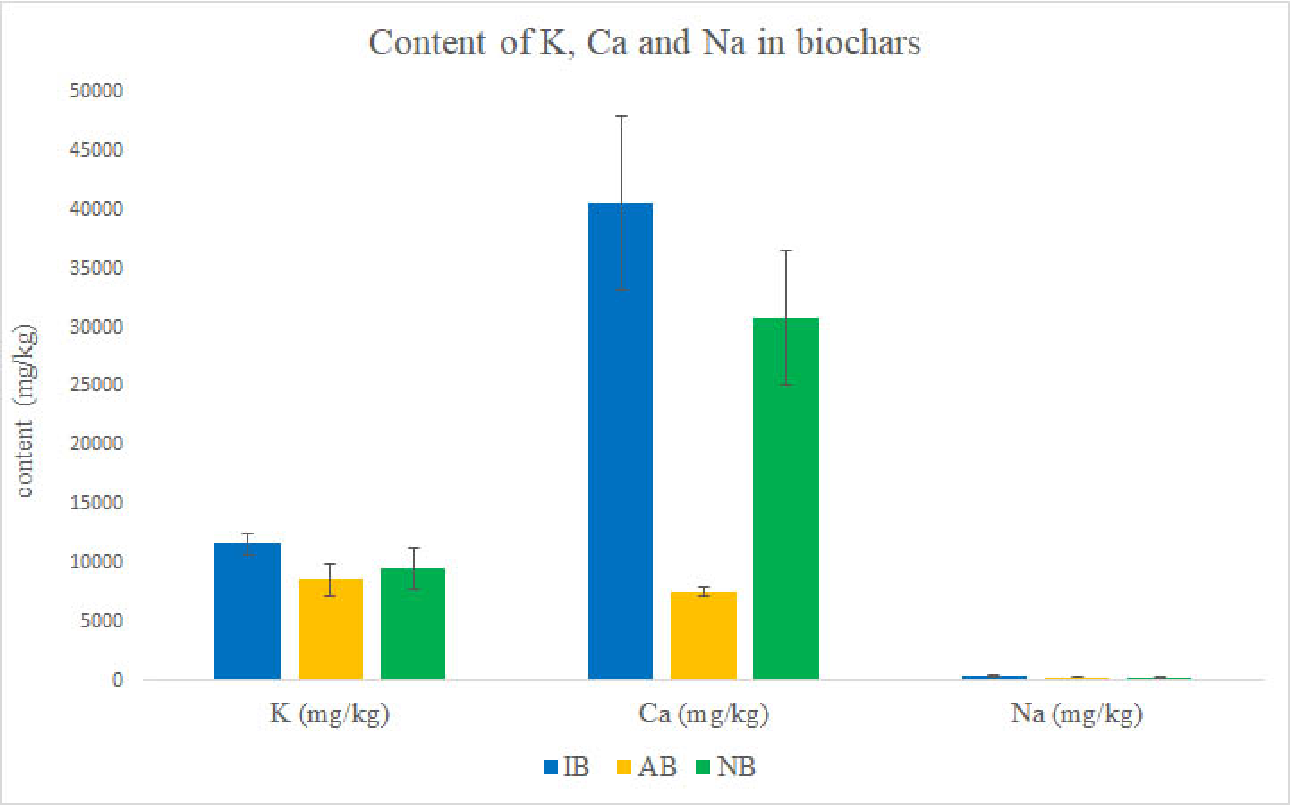

Figure S4: Content of potassium (K), calcium (Ca) and sodium (Na) in biochar; Abbreviations: IB—"Italian" biochar; AB—Aries Green biochar; NB-Naked biochar.

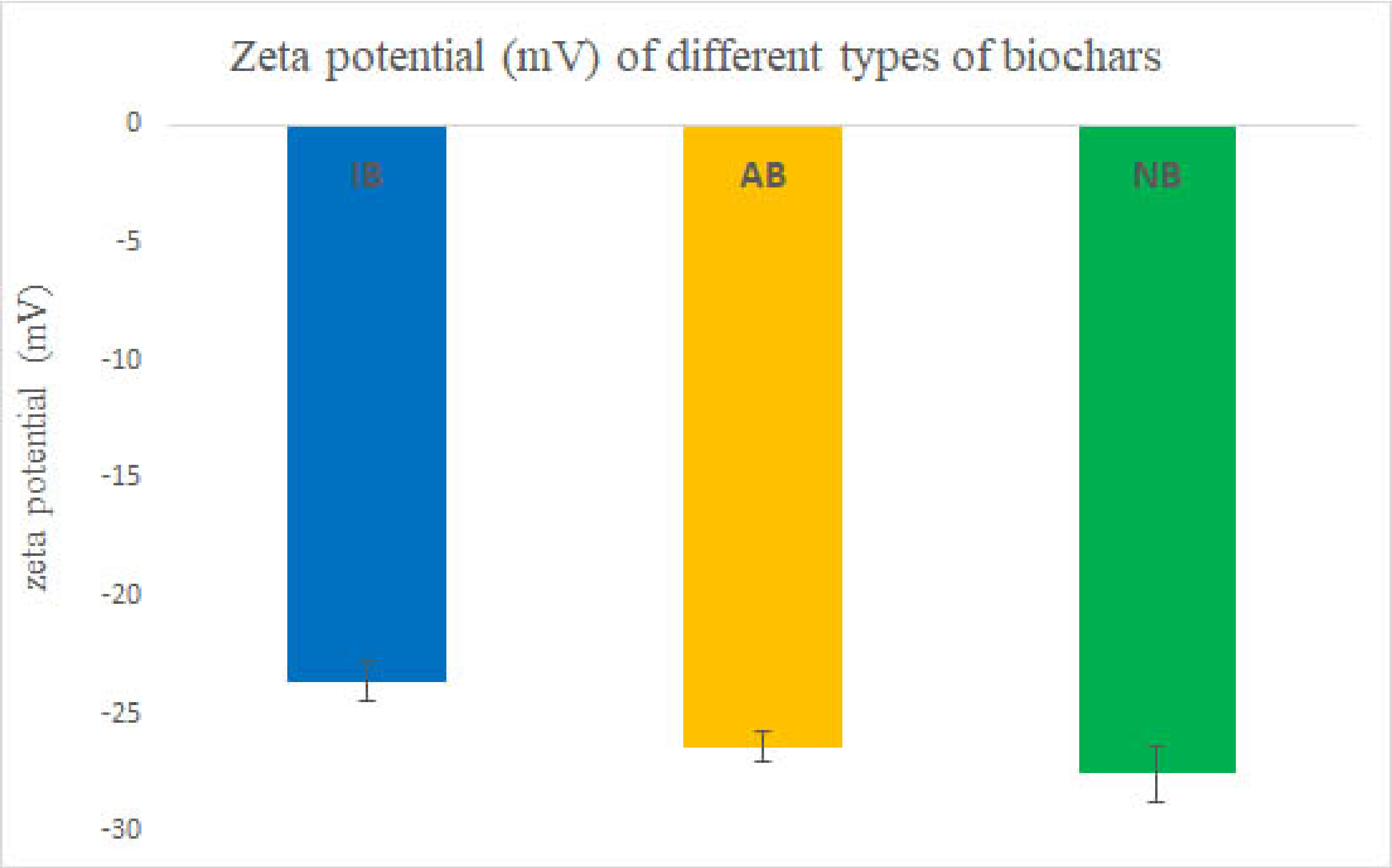

Figure S5: ζ potential of biochars. Abbreviations: IB—"Italian" biochar; AB—Aries Green biochar; NB-Naked biochar.

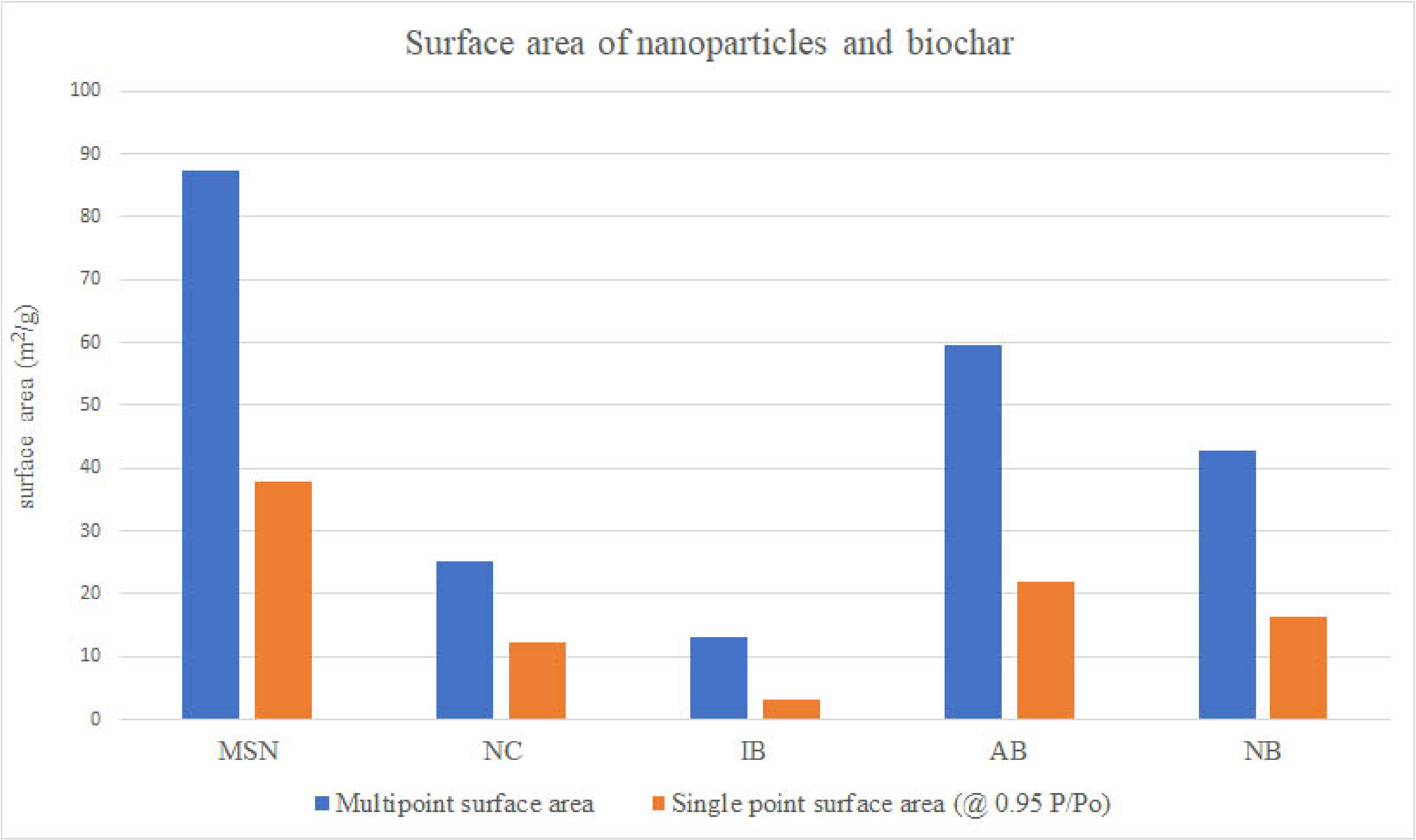

Figure S6: Surface area of nanoparticles and biochars. Abbreviations: MSN—chitosan coated mesoporous silica; NC—nanoclay; IB—"Italian" biochar; AB—Aries Green biochar; NB-Naked biochar.

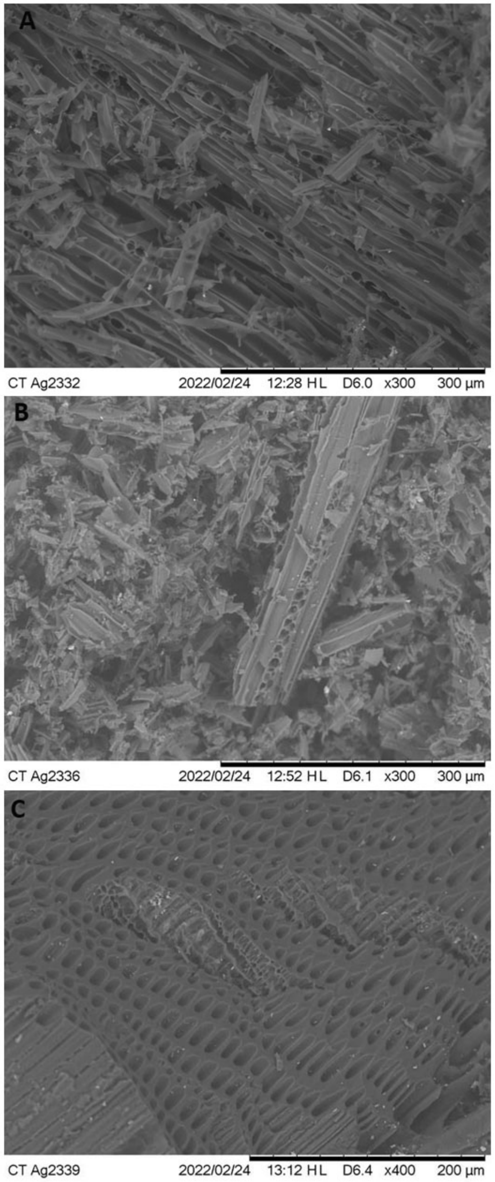

Figure S7: Representative scanning electron microscopy (SEM) images of biochars. A—“Italian” biochar; B—Aries Green biochar; C-Naked biochar.

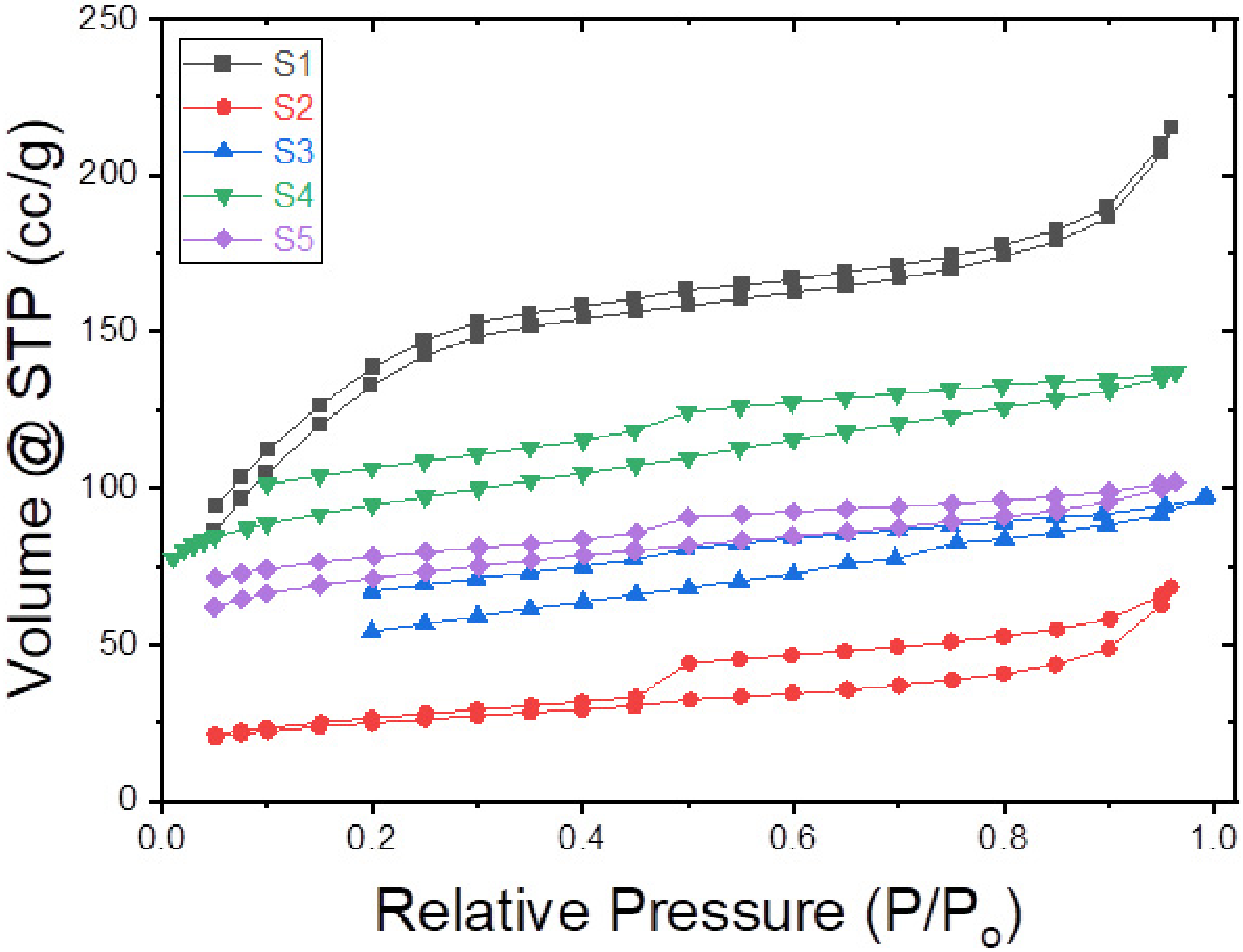

Figure S8: Pore volume of nanoparticles and biochars. Abbreviations: S1—chitosan coated mesoporous silica; S2—nanoclay; S3—"Italian" biochar; S4—Aries Green biochar; S5—Naked biochar.

Table S4: Content of phosphorus (P) and nitrogen (N) in biochars*

sample	P content (mg/kg)	N content (mg/g)	P/N (mg/mg)
Aries Green biochar (AB)	363.3 ± 13.7[a]	7.5 ± 0.6[a]	0.05
Naked biochar (NB)	4163.3 ± 304.6[b]	3.9 ± 0.4[b]	1.07
"Italian" biochar (IB)	21703.6 ± 2813.5[c]	5.8 ± 0.2[c]	3.74

*Different letters in the same column means that differences between samples were statistically significant (determined by Tukey test at $p < 0.05$)

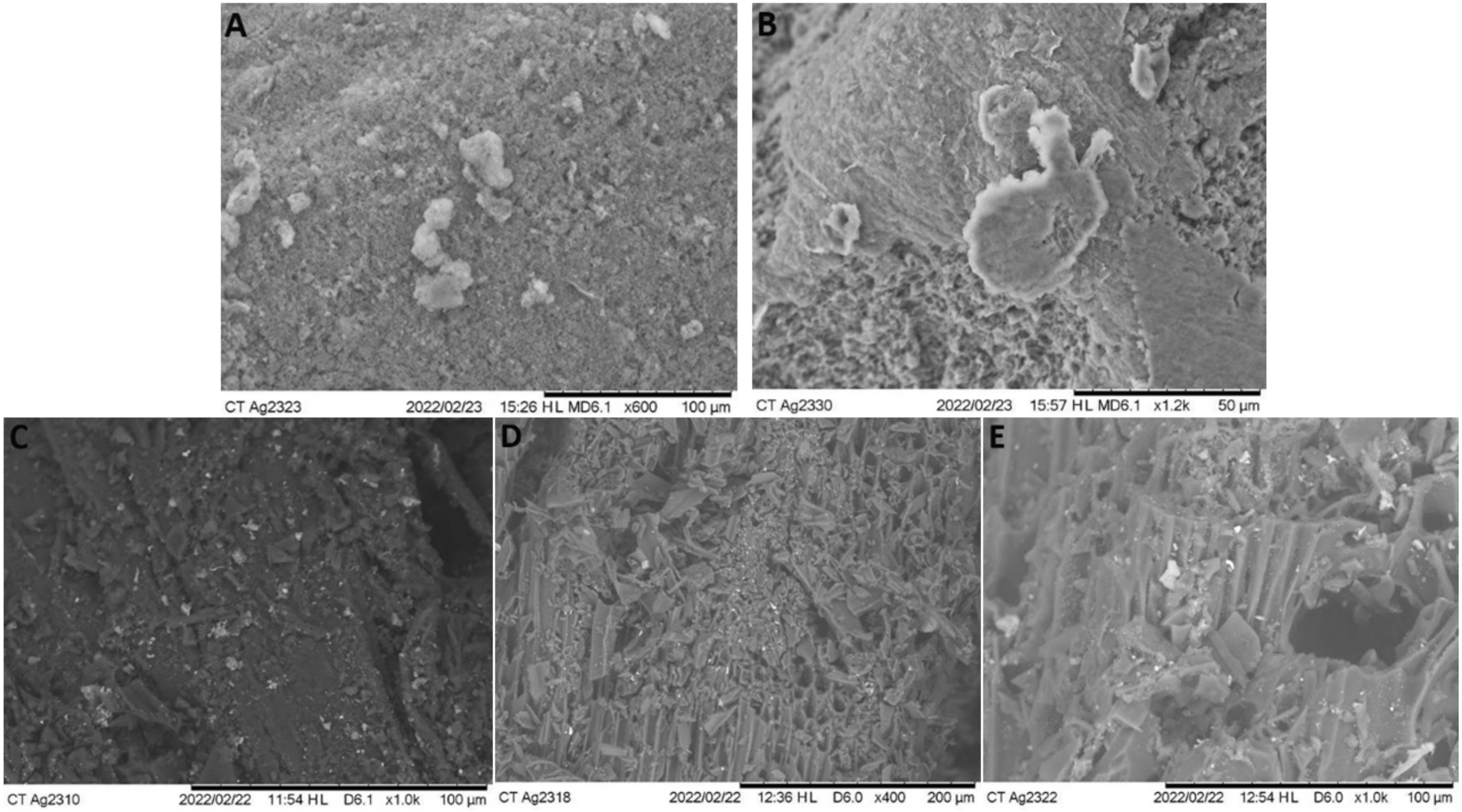

Figure S9: Representative scanning electron microscopy (SEM) images of nanoparticles and biochars with adsorbed bacteria. A—chitosan-coated mesoporous silica + bacteria; B—nanoclay + bacteria; C—“Italian” biochar + bacteria; D—Aries Green biochar + bacteria; E—Naked biochar + bacteria.

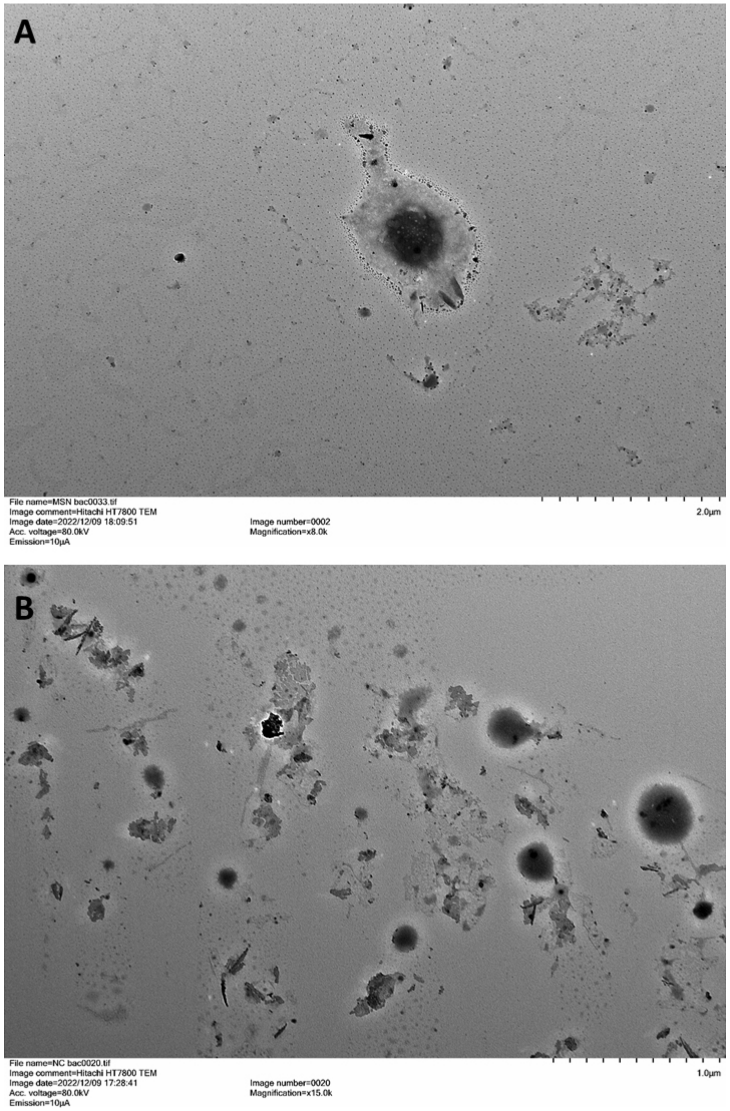

Figure S10: Representative transmission electron microscopy (TEM) images of nanoparticles with adsorbed bacteria. A—chitosan-coated mesoporous silica + bacteria; B—nanoclay + bacteria.

Table S5: Content of P and N in soil substrate and fertilizer

sample	P content (mg/kg)	N content (mg/kg)
Soil substrate (Promix)	562.32 ± 92.96	0. 23 ± 0.02
Fertilizer (Miracle-Gro)	37822.97 ± 1083.65	22.95 ± 2.22

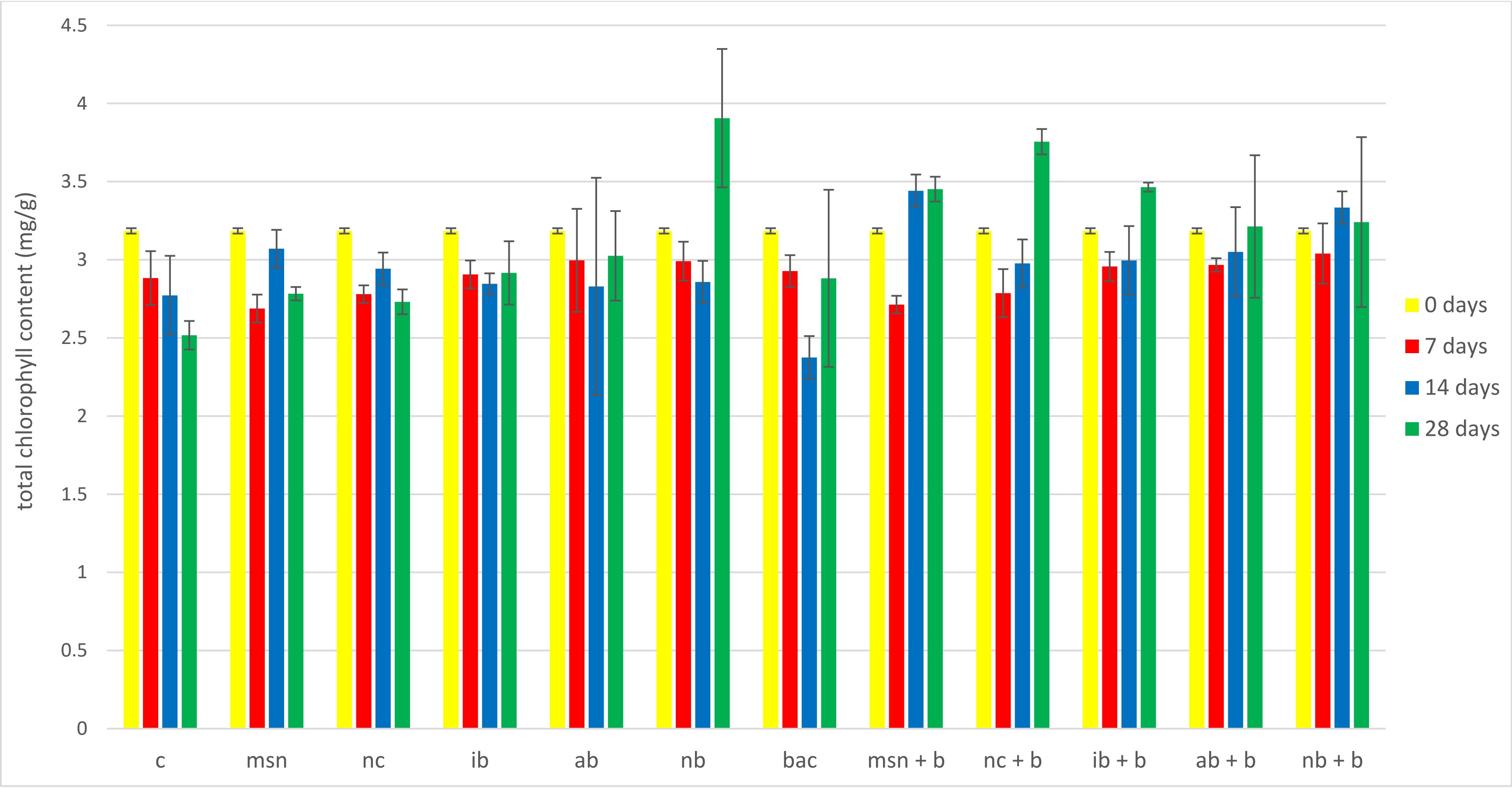

Figure S11: Total chlorophyll content (mg/g fresh mass) in 1st tomato experiment. Abbreviations: C—control; MSN—chitosan-coated mesoporous silica nanoparticles; NC—nanoclay; IB—"Italian" biochar; AB—Aries Green biochar; NB—Naked biochar; B—bacteria; MSN + B—chitosan-coated mesoporous silica nanoparticles with adsorbed bacteria; NC + B—nanoclay with adsorbed bacteria; IB + B—"Italian" biochar with adsorbed bacteria; AB + B—Aries Green biochar with adsorbed bacteria; NB + B—Naked biochar with adsorbed bacteria.

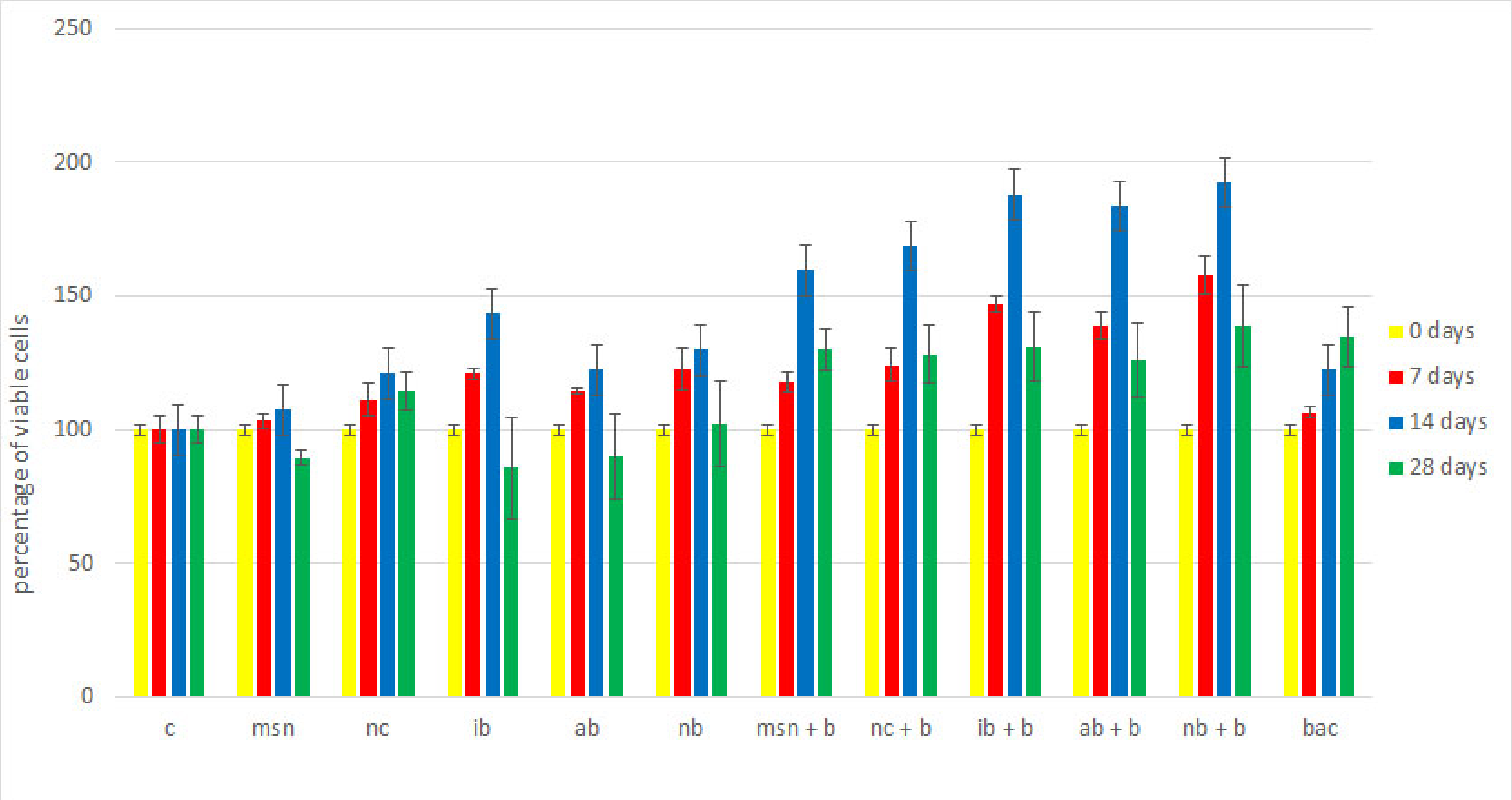

Figure S12: Cell viability in 1st tomato experiment. Abbreviations: C—control; MSN—chitosan-coated mesoporous silica nanoparticles; NC—nanoclay; IB—“Italian” biochar; AB—Aries Green biochar; NB—Naked biochar; B—bacteria; MSN + B—chitosan-coated mesoporous silica nanoparticles with adsorbed bacteria; NC + B—nanoclay with adsorbed bacteria; IB + B—“Italian” biochar with adsorbed bacteria; AB + B—Aries Green biochar with adsorbed bacteria; NB + B—Naked biochar with adsorbed bacteria.

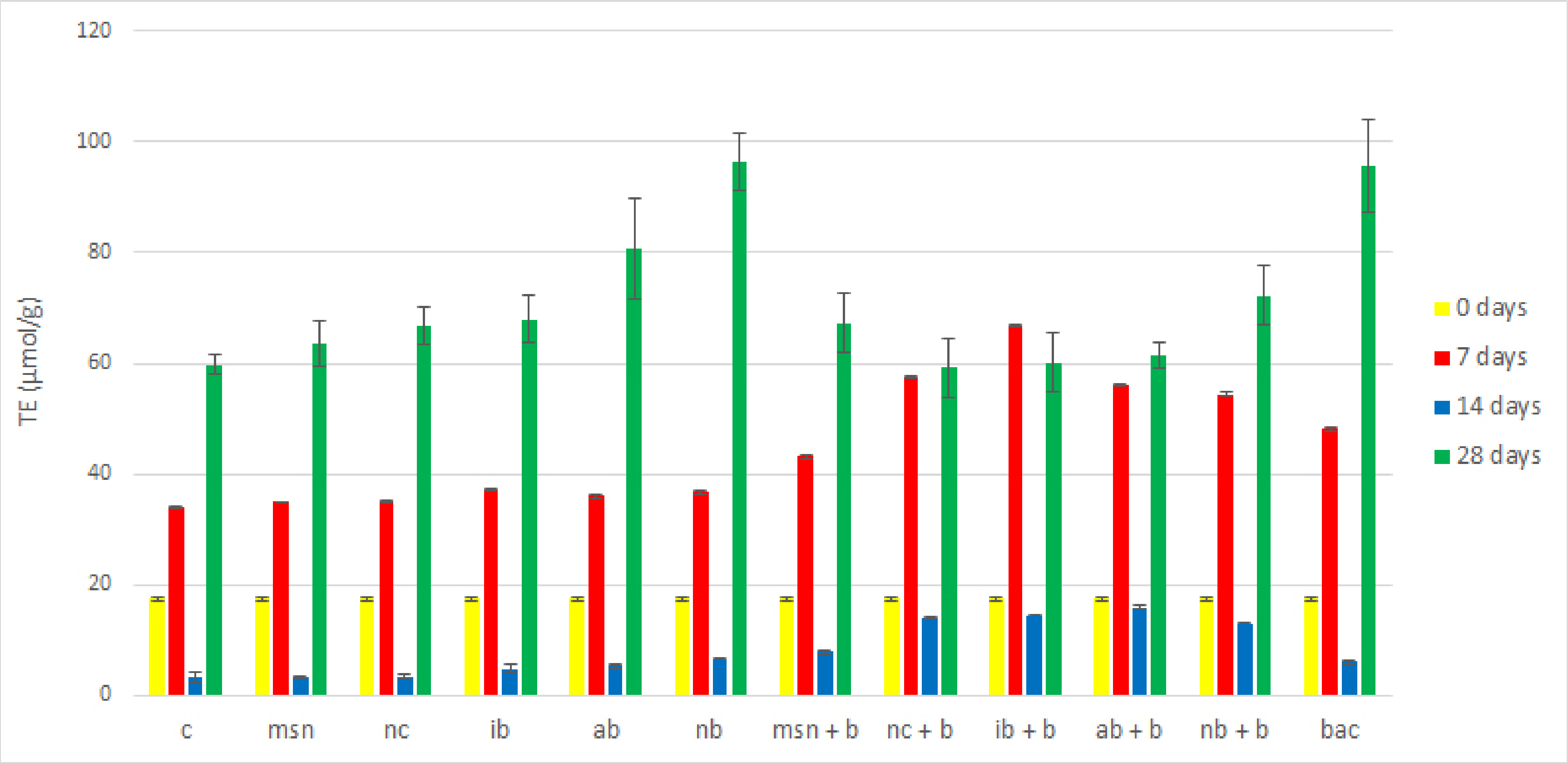

Figure S13: Antioxidative properties in thein 1st tomato experiment measured by DPPH. Abbreviations: C—control; MSN—chitosan-coated mesoporous silica nanoparticles; NC—nanoclay; IB—"Italian" biochar; AB—Aries Green bio-char; NB—Naked biochar; B—bacteria; MSN + B—chitosan-coated mesoporous silica nanoparticles with adsorbed bacteria; NC + B—nanoclay with adsorbed bacteria; IB + B—"Italian" biochar with adsorbed bacteria; AB + B—Aries Green biochar with adsorbed bacteria; NB + B—Naked biochar with adsorbed bacteria.

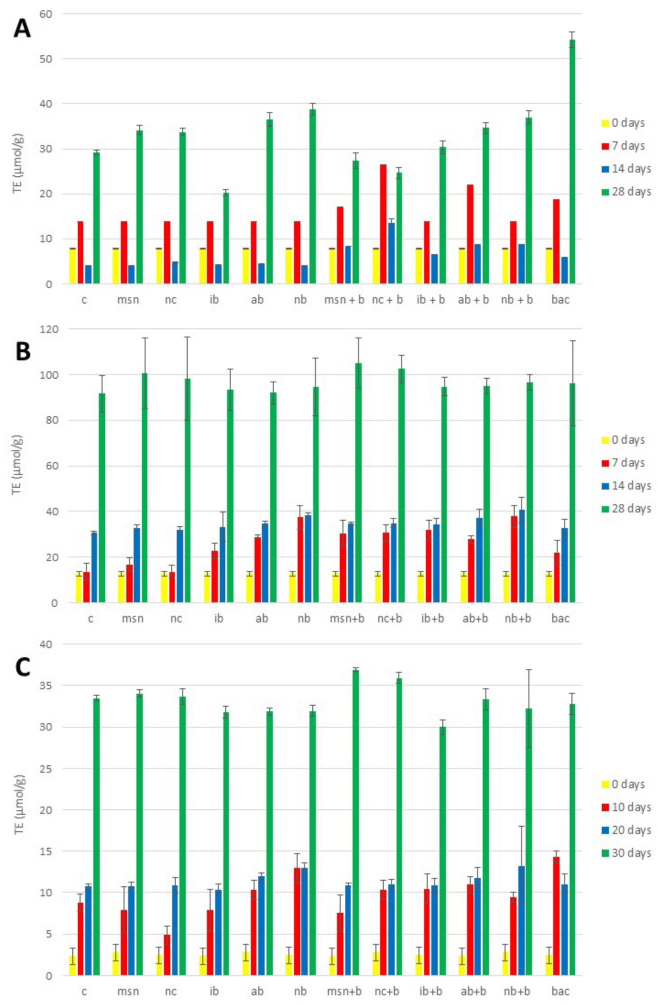

Figure S14: Antioxidative properties measured by ABTS assay. A—1st tomato experiment; B—2nd tomato experiment; C—watermelon experiment. Abbreviations: C—control; MSN—chitosan-coated mesoporous silica nanoparticles; NC—nanoclay; IB—"Italian" biochar; AB—Aries Green biochar; NB—Naked biochar; BAC—bacteria; MSN + B—chitosan-coated mesoporous silica nanoparticles with adsorbed bacteria; NC + B—nanoclay with adsorbed bacteria; IB + B—"Italian" biochar with adsorbed bacteria; AB + B—Aries Green biochar with adsorbed bacteria; NB + B—Naked biochar with adsorbed bacteria.

Table S6: Changes in soil pH in 1st tomato experiment, 2nd tomato experiment and watermelon experiment*

sample	1st tomato experiment				2nd tomato experiment				watermelon experiment			
	0 days	7 days	14 days	28 days	0 days	7 days	14 days	28 days	0 days	10 days	20 days	30 days
C	6.3±0.1^{a}	6.3±0.1^{a}	6.3±0.2^{a}	6.5±0.1^{a}	5.7±0.1^{a}	5.8±0.1^{a}	6.0±0.1^{a}	6.2±0.1^{a}	5.7±0.1^{a}	6.2±0.1^{a}	6.3±0.2^{a}	6.4±0.2^{a}
MSN	6.3±0.1^{a}	6.4±0.1^{a}	6.6±0.1^{b}	6.6±0.1^{b}	5.7±0.1^{a}	5.9±0.1^{a,b}	6.0±0.1^{a}	6.2±0.1^{a}	5.7±0.1^{a}	6.4±0.2^{a}	6.6±0.1^{b}	6.0±0.2^{b}
NC	6.3±0.1^{a}	6.2±0.2^{a}	6.4±0.2^{a}	6.5±0.2^{a}	5.7±0.1^{a}	5.9±0.1^{a,b}	5.9±0.1^{a,b}	6.1±0.1^{a}	5.7±0.1^{a}	6.2±0.2^{a}	6.2±0.1^{a}	5.8±0.1^{b}
IB	6.3±0.1^{a}	6.4±0.1^{a}	6.5±0.1^{a}	6.7±0.1^{a}	5.7±0.1^{a}	5.9±0.1^{a,b}	5.9±0.1^{a,b}	6.2±0.1^{a}	5.7±0.1^{a}	6.2±0.1^{a}	6.1±0.1^{a}	5.9±0.2^{b}
AB	6.3±0.1^{a}	6.5±0.2^{a}	6.5±0.1^{b}	6.7±0.1^{a}	5.7±0.1^{a}	5.8±0.1^{a}	6.0±0.1^{a}	6.0±0.1^{a,b}	5.7±0.1^{a}	6.2±0.1^{a}	6.0±0.1^{a}	5.8±0.1^{b}
NB	6.3±0.1^{a}	6.6±0.1^{b}	6.5±0.1^{a}	6.7±0.1^{a}	5.7±0.1^{a}	5.9±0.1^{a,b}	5.9±0.1^{a,b}	6.2±0.1^{a}	5.7±0.1^{a}	6.4±0.2^{a}	6.3±0.2^{a}	6.0±0.2^{b}
BAC	6.3±0.1^{a}	6.8±0.2^{a}	6.5±0.2^{a}	6.8±0.1^{c}	5.7±0.1^{a}	5.8±0.1^{a}	6.0±0.1^{a}	6.4±0.1^{a,c}	5.7±0.1^{a}	6.2±0.1^{a}	6.3±0.2^{a}	6.0±0.2^{a,b}
MSN + B	6.3±0.1^{a}	6.7±0.1^{c}	6.7±0.1^{c}	6.7±0.1^{c}	5.7±0.1^{a}	6.1±0.1^{b}	6.0±0.1^{a}	6.1±0.1^{a}	5.7±0.1^{a}	6.2±0.1^{a}	6.2±0.2^{a}	5.9±0.1^{b}
NC + B	6.3±0.1^{a}	6.6±0.1^{b}	6.8±0.1^{b}	6.8±0.2^{a}	5.7±0.1^{a}	6.1±0.1^{b}	6.0±0.1^{a}	5.9±0.1^{b}	5.7±0.1^{a}	6.4±0.2^{a}	6.0±0.1^{a}	5.9±0.1^{b}
IB + B	6.3±0.1^{a}	6.5±0.2^{a}	6.6±0.2^{b}	6.8±0.2^{a}	5.7±0.1^{a}	5.8±0.1^{a}	6.1±0.1^{a}	6.1±0.1^{a}	5.7±0.1^{a}	6.2±0.1^{a}	6.2±0.1^{a}	6.0±0.1^{b}
AB + B	6.3±0.1^{a}	6.8±0.1^{c}	6.7±0.1^{b}	6.8±0.2^{a}	5.7±0.1^{a}	5.8±0.1^{a}	6.2±0.2^{a}	6.0±0.1^{a,b}	5.7±0.1^{a}	6.2±0.1^{a}	6.2±0.1^{a}	6.0±0.1^{b}
NB + B	6.3±0.1^{a}	6.7±0.1^{b}	6.8±0.1^{b}	6.7±0.2^{a}	5.7±0.1^{a}	5.9±0.1^{a,b}	5.9±0.1^{a,b}	5.9±0.1^{b}	5.7±0.1^{a}	6.1±0.1^{a}	6.1±0.1^{a}	5.8±0.1^{b}

*Different letters in the same column means that differences between samples were statistically significant (determined by Tukey test at $p < 0.05$). Abbreviations: C – control; MSN – chitosan-coated mesoporous silica nanoparticles; NC – nanoclay; IB – "Italian" biochar; AB – Aries Green biochar; NB – Naked biochar; BAC - bacteria; MSN + B – chitosan-coated mesoporous silica nanoparticles with adsorbed bacteria; NC + B – nanoclay with adsorbed bacteria; IB +B – "Italian" biochar with adsorbed bacteria; AB +B – Aries Green biochar with adsorbed bacteria; NB +B –Naked biochar with adsorbed bacteria

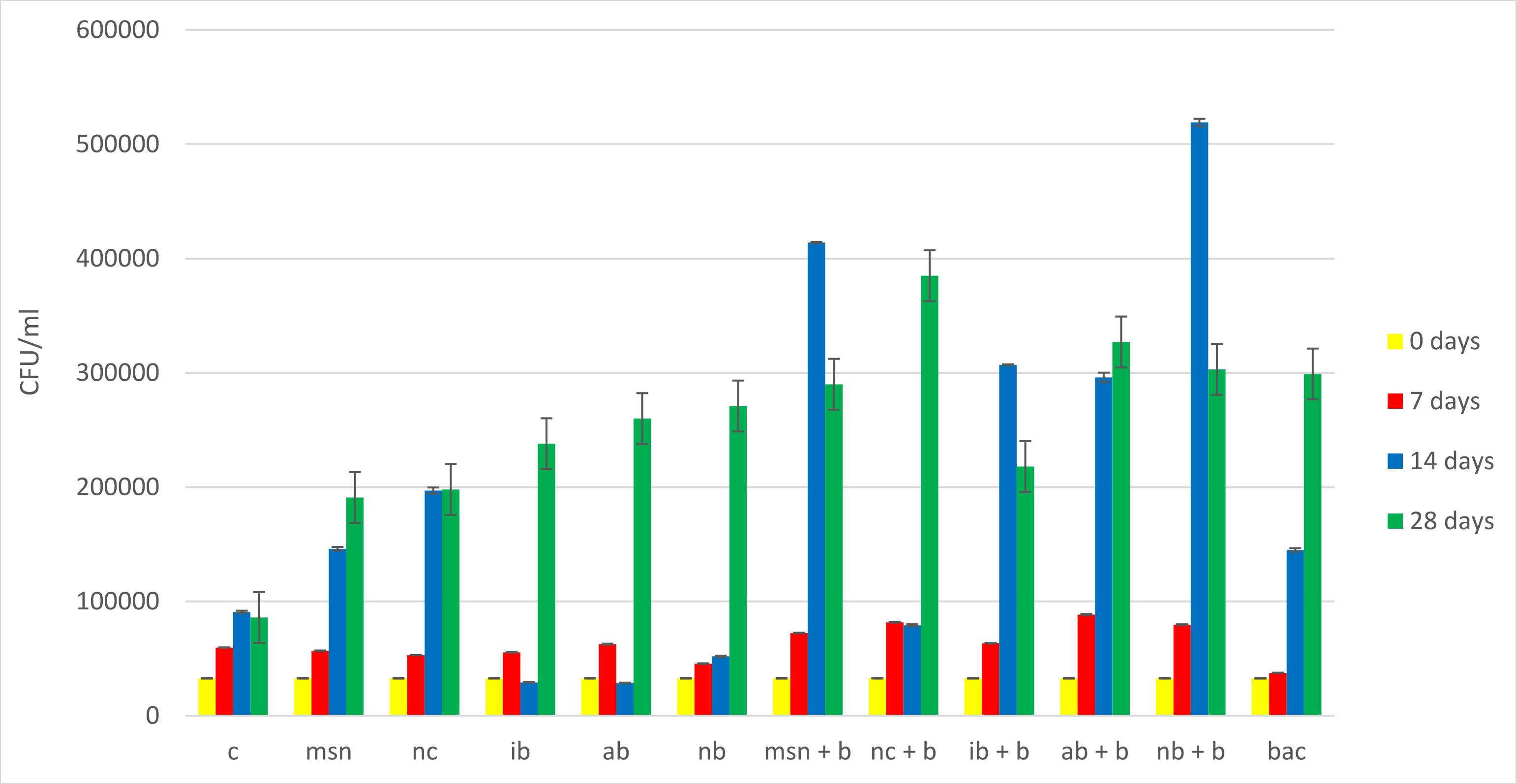

Figure S15: Content of total bacteria in the soil in 1st tomato experiment. Abbreviations: C—control; MSN—chitosan-coated mesoporous silica nanoparticles; NC—nanoclay; IB—"Italian" biochar; AB—Aries Green bio-char; NB—Naked biochar; B—bacteria; MSN + B—chitosan-coated mesoporous silica nanoparticles with adsorbed bacteria; NC + B—nanoclay with adsorbed bacteria; IB + B—"Italian" biochar with adsorbed bacteria; AB + B—Aries Green biochar with adsorbed bacteria; NB + B—Naked biochar with adsorbed bacteria.

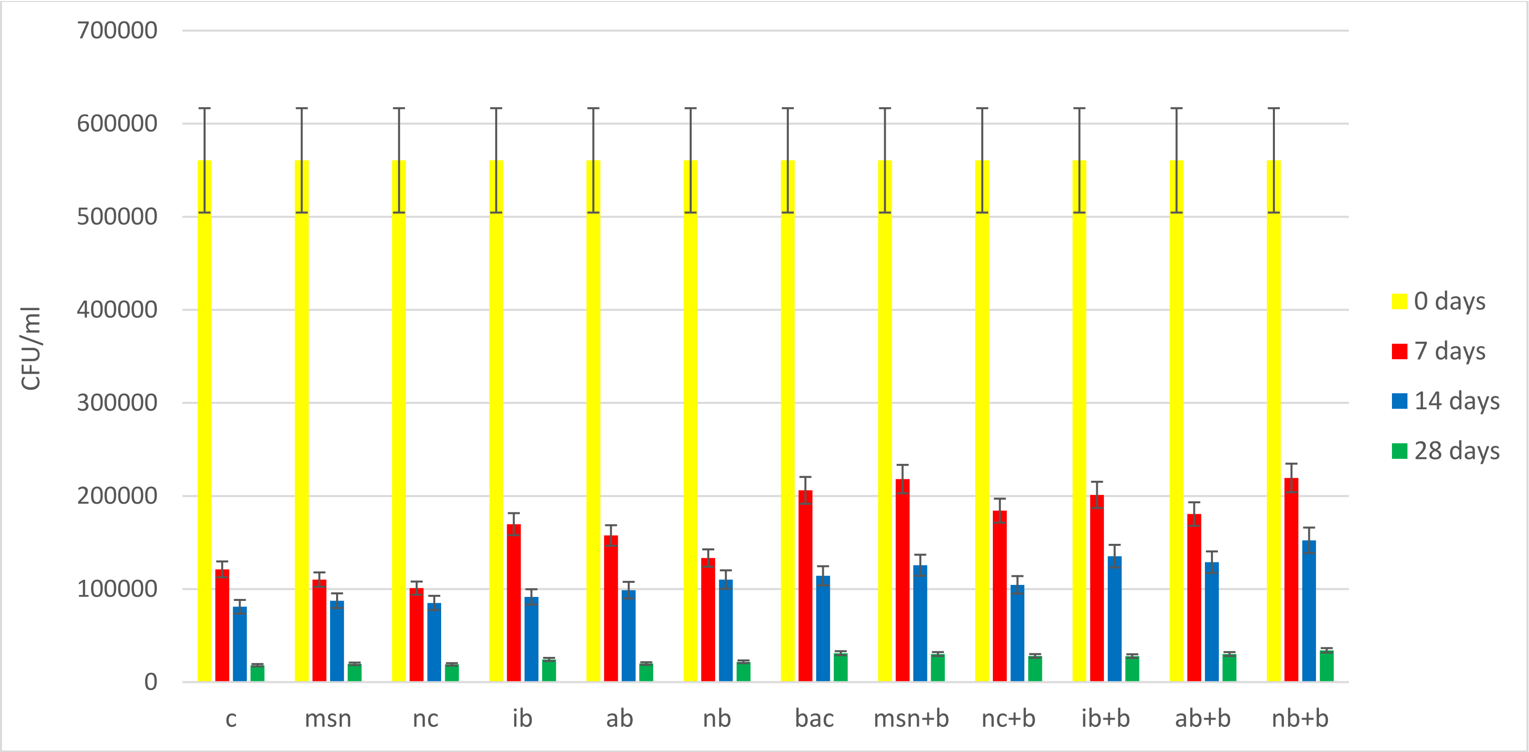

Figure S16: Content of nitrogen-fixing bacteria in the soil in 1st tomato experiment. Abbreviations: C—control; MSN—chitosan-coated mesoporous silica nanoparticles; NC—nanoclay; IB—"Italian" biochar; AB—Aries Green bio-char; NB—Naked biochar; B—bacteria; MSN + B—chitosan-coated mesoporous silica nanoparticles with adsorbed bacteria; NC + B—nanoclay with adsorbed bacteria; IB + B—"Italian" biochar with adsorbed bacteria; AB + B—Aries Green biochar with adsorbed bacteria; NB + B—Naked biochar with adsorbed bacteria.

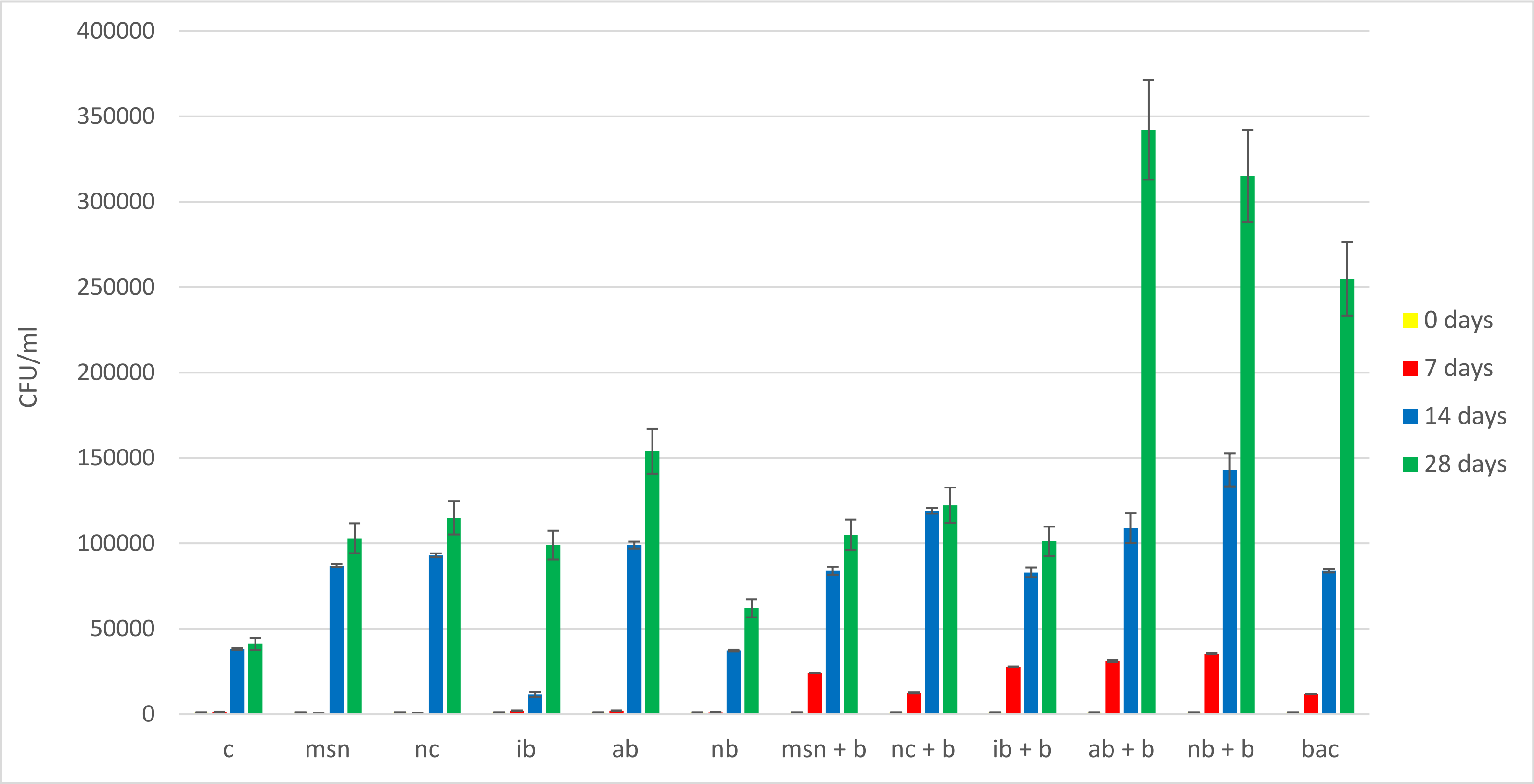

Figure S17: Content of phosphorus-solubilizing bacteria in 1st tomato experiment. Abbreviations: C—control; MSN—chitosan-coated mesoporous silica nanoparticles; NC—nanoclay; IB—"Italian" biochar; AB—Aries Green biochar; NB—Naked biochar; B—bacteria; MSN + B—chitosan-coated mesoporous silica nanoparticles with adsorbed bacteria; NC + B—nanoclay with adsorbed bacteria; IB + B—"Italian" biochar with adsorbed bacteria; AB + B—Aries Green biochar with adsorbed bacteria; NB + B—Naked biochar with adsorbed bacteria.
